# Transcriptomic Analyses Reveal Differential Gene Expression of Immune and Cell Death Pathways in the Brains of Mice Infected with West Nile Virus and Chikungunya Virus

**DOI:** 10.3389/fmicb.2017.01556

**Published:** 2017-08-17

**Authors:** Stephanie M. Lim, Henk-Jan van den Ham, Minoushka Oduber, Eurydice Martina, Fatiha Zaaraoui-Boutahar, Jeroen M. Roose, Wilfred F. J. van IJcken, Albert D. M. E. Osterhaus, Arno C. Andeweg, Penelope Koraka, Byron E. E. Martina

**Affiliations:** ^1^Artemis One Health Research Foundation Delft, Netherlands; ^2^Department of Viroscience, Erasmus Medical Center Rotterdam, Netherlands; ^3^Center for Biomics, Erasmus Medical Center Rotterdam, Netherlands; ^4^Research Center for Emerging Infections and Zoonoses (RIZ), University of Veterinary Medicine Hannover, Germany

**Keywords:** transcriptomics, Genomics, West Nile virus, chikungunya virus, neuroinvasive disease, cell death mechanisms, immune response

## Abstract

West Nile virus (WNV) and chikungunya virus (CHIKV) are arboviruses that are constantly (re-)emerging and expanding their territory. Both viruses often cause a mild form of disease, but severe forms of the disease can consist of neurological symptoms, most often observed in the elderly and young children, respectively, for which the mechanisms are poorly understood. To further elucidate the mechanisms responsible for end-stage WNV and CHIKV neuroinvasive disease, we used transcriptomics to compare the induction of effector pathways in the brain during the early and late stage of disease in young mice. In addition to the more commonly described cell death pathways such as apoptosis and autophagy, we also found evidence for the differential expression of pyroptosis and necroptosis cell death markers during both WNV and CHIKV neuroinvasive disease. In contrast, no evidence of cell dysfunction was observed, indicating that cell death may be the most important mechanism of disease. Interestingly, there was overlap when comparing immune markers involved in neuroinvasive disease to those seen in neurodegenerative diseases. Nonetheless, further validation studies are needed to determine the activation and involvement of these effector pathways at the end stage of disease. Furthermore, evidence for a strong inflammatory response was found in mice infected with WNV and CHIKV. The transcriptomics profile measured in mice with WNV and CHIKV neuroinvasive disease in our study showed strong overlap with the mRNA profile described in the literature for other viral neuroinvasive diseases. More studies are warranted to decipher the role of cell inflammation and cell death in viral neuroinvasive disease and whether common mechanisms are active in both neurodegenerative and brain infectious diseases.

## Introduction

Over the past decades, arboviruses such as West Nile virus (WNV) and chikungunya virus (CHIKV) have emerged in multiple countries across different continents, illustrating their potential to pose a global public health threat. Although infections with these viruses are usually benign, severe forms of WNV and CHIKV infections may include neurologic manifestations that are not well-understood. WNV and CHIKV are arthropod-borne, enveloped, positive-sense RNA viruses that belong to the family *Flaviviridae* and *Togaviridae*, respectively. WNV is maintained in an enzootic transmission cycle comprising birds, horses, and humans. Birds act as amplification hosts and transmit the virus to mosquitoes, predominantly *Culex* species, while humans and horses serve as incidental dead-end hosts (Rossi et al., 2010). The transmission cycle of CHIKV is mainly an urban mosquito-human cycle involving anthropophilic *Aedes* mosquitoes; *Aedes aegypti* and *Aedes albopictus* (Burt et al., 2012), but in West-Africa CHIKV is maintained in a sylvatic cycle by sylvatic forest-dwelling mosquitoes (Diallo et al., 1999).

Although human infections with WNV are asymptomatic in the majority of cases, 20–30% may develop West Nile fever, a mild flu-like illness consisting of symptoms such as malaise, eye pain, headache, myalgia, gastrointestinal discomfort, and rash. However, 1% of persons with clinical illness may develop neuroinvasive disease such as meningitis, encephalitis, and acute flaccid paralysis (AFP) (Campbell et al., 2002; Petersen and Marfin, 2002), and long-term neurological sequelae, such as persistent tremors and Parkinson-like disease, may present in more than 50% of these cases. Patients with compromised immune systems, the elderly, children, and people with underlying conditions such as diabetes mellitus are especially at risk of developing severe disease (Sejvar, 2014).

Clinical symptoms of CHIKV infection are characterized by fever and rash, followed by myalgia and arthralgia (Dupuis-Maguiraga et al., 2012). Chikungunya fever is rarely fatal with symptoms resolving in weeks, but some patients suffer from joint pain in the form of recurrent or persistent episodes that can last for months to years (Ali Ou Alla and Combe, 2011). Severe clinical manifestations of CHIKV infection often involve the central nervous system (CNS) and occur most notably, though not exclusively, in young children and the elderly, and were commonly reported during the outbreaks in La Réunion (Tournebize et al., 2009; Gerardin et al., 2016) and India (Chandak et al., 2009; Lewthwaite et al., 2009; Peter et al., 2015).

WNV replicates in a wide variety of cell types and *in vitro* and *in vivo* studies suggest that neurons and astrocytes in the CNS are targeted (Shrestha et al., 2003; Cheeran et al., 2005; Diniz et al., 2006; Hussmann et al., 2013; Lim et al., 2013). In contrast, it is not clear whether CHIKV has tropism for neurons and it has been speculated that the neurological symptoms are instead the result of infection of the choroid plexus and meninges (Couderc et al., 2008). Studies using adult immunosuppressed AG129 mice did not demonstrate infection of neurons (van den Doel et al., 2014), while studies using adult severely immunosuppressed NRG mice did show infection of neurons (Poo et al., 2014). In addition, *in vitro* studies have shown that CHIKV infects both astrocytes and neurons, while astrocytes were targeted in the brain of OF1 newborn mice (Das et al., 2015). Nonetheless, factors governing the development of an immune response in the brain and the causes of neurologic disease in both WNV- and CHIKV-infected patients are poorly understood.

Even though the pathogenesis of WNV neuroinvasive disease has not been fully elucidated, several studies have provided evidence that apoptosis plays a central role during infection with this virus *in vitro* and in mice (Parquet et al., 2001; Yang et al., 2002, 2008; Chu and Ng, 2003; Kleinschmidt et al., 2007; Samuel et al., 2007; Clarke et al., 2014b). CHIKV has also been shown to induce apoptosis *in vitro* (Krejbich-Trotot et al., 2011a; Nayak et al., 2017) and *in vivo* (Joubert et al., 2012), including in the brain (Chiam et al., 2015) or in cells derived from the brain (Dhanwani et al., 2012; Abraham et al., 2013). However, little is known about the involvement of other programmed cell death pathways, such as pyroptosis and necroptosis, in the pathogenesis of WNV or CHIKV neuroinvasive disease. As programmed cell death is a genetically controlled process, this suggests that unraveling the mechanisms involved may support the design of novel treatment protocols for WNV or CHIKV neuroinvasive disease.

Apart from programmed cell death, conceptually, cellular dysfunction may also contribute to the pathogenesis of neuroinvasive disease. Cellular dysfunction is frequently the result of mitochondrial dysfunction in which a deficit in energy metabolism can damage the entire physiology of an organism (Schon and Manfredi, 2003). Although progressive neuronal loss most likely contributes to neurologic manifestations in humans presenting with WNV neuroinvasive disease (Tselis and Booss, 2014), it is possible that long-term sequelae may also reflect dysfunction rather than loss of neurons. To date, cellular dysfunction pathways have not yet been investigated for WNV.

The changing epidemiology of WNV and CHIKV, as well as the constant risk of (re-)emerging virulent strains, highlight the importance of understanding the underlying mechanisms of severe WNV and CHIKV disease. As these mechanisms can only be studied to a limited extent in human patients, the mouse model is frequently used to study effector pathways involved in meningitis/encephalitis. In addition, studying WNV and CHIKV neurologic disease in a mouse model can help unravel the effector mechanisms involved in viral pathogenesis, such as cell dysfunction and/or cell death. Furthermore, comparing inflammatory response markers during the late stage of WNV and CHIKV neuroinvasive disease and those seen in neurodegenerative diseases might help to unravel the possible etiology of neurologic diseases, as well as potential common treatments.

A sensitive tool for investigating pathogenesis is transcriptome analysis, which has been used here to examine gene expression changes as a result of WNV and CHIKV infection, and to identify pathways that may be affected. Several studies have been conducted that examine gene expression upon WNV infection, such as in the brains of mice (Venter et al., 2005; Clarke et al., 2014a; Kumar et al., 2016), in the thalamus and cerebrum of experimentally infected horses (Bourgeois et al., 2011), as well as in human retinal pigment epithelium (hRPE) cells (Munoz-Erazo et al., 2012). To date, however, no such studies have been carried out for CHIKV.

In the present study, we have conducted a global transcriptional analysis of the mouse brain in response to WNV and CHIKV infection, in which we compared transcriptome snapshots from animals with early and severe neuroinvasive disease to identify putative pathways and mechanisms involved in the end stage of WNV and CHIKV pathogenesis. Furthermore, we compared our findings to results from other studies that conducted gene expression analyses in the brain after WNV infection.

## Materials and methods

### Cells and virus

Vero E6 cells were grown in DMEM (Lonza Benelux BV, Breda, the Netherlands) supplemented with antibiotics (100 U penicillin, 100 μg/mL of streptomycin), 10% heat inactivated fetal calf serum (FCS), 0.75% sodium bicarbonate, and 10 mM hepes buffer (all from Lonza). Mice were infected with WNV strain NY99 (accession AF196835.2, obtained from the Health Protection Agency, Porton Down, UK; P5 on Vero E6 cells) or CHIKV strain S27, accession AF369024). The 50% tissue culture infectious dose (TCID_50_) was determined on Vero E6 cells using the Spearman & Kärber method based on the presence of cytopathic effects 5 days post inoculation (Lim et al., 2013).

### Mouse infection

Nine-day old female C57/BL6 mice (Harlan Laboratories B.V., Venray, The Netherlands) were inoculated intraperitoneally (i.p.) with either 10^5^ TCID_50_/100 μL of WNV-NY99 (*n* = 6) or 10^6.5^ TCID_50_/100 μL of CHIKV-S27 (*n* = 6). Mice were sacrificed at the indicated time points (see Results Section) in order to determine the kinetics of neuroinvasive disease and perform transcriptomic analysis. For the kinetics experiment, brains were collected in 1 ml of DMEM containing antibiotics (100 U penicillin, 100 μg/mL streptomycin) (right hemisphere) or formalin (left hemisphere). For transcriptomic analysis, cerebellum was separated from the brain and the right hemisphere was gently washed once in ice-cold phosphate buffered saline (PBS) and stored in RNAlater® (Thermo Fisher Scientific, Bleiswijk, The Netherlands) until further use.

In order to generalize the results obtained in young animals to adult mice, female 6-week old C57/BL6 mice were infected i.p. with 10^4^ TCID_50_/100 μL of WNV-NY99 (*n* = 8). Mice were euthanized when humane endpoints were reached (immobility and paralysis) between days 9 and 12 p.i. In addition, six animals were inoculated with DMEM (control group) and euthanized on day 2 after inoculation. Brains were collected and the right hemisphere was stored in 1 mL of DMEM containing antibiotics and stored at −80°C until further processing.

Mice were maintained in isolator cages throughout the infection experiment, had a 12 h day-night cycle and were fed *ad libitum*. Animal experiments were approved by the Animal Ethics Committee of Erasmus Medical Center under protocol number 122–11–01. Animals were euthanized by cervical dislocation under isoflurane anesthesia.

### Quantitation of virus in the brain

Half-brains in virus transport medium were homogenized and RNA was isolated from 100 μL of homogenized tissue using the MagNA Pure LC Total Nucleic Acid Isolation kit (Roche, Almere, The Netherlands) and an automated nucleic acid robotic workstation (Roche) according to the manufacturer's instructions. RNA copy numbers in the brain were determined using the TaqMan® EZ RT-PCR kit (Applied Biosystems, Bleiswijk, The Netherlands) employing primers and probe located on the 3′UTR for WNV, as previously described in Lim et al. (2013), or primers and probe located on the E1 protein for CHIKV (van den Doel et al., 2014). Viral RNA load was determined from a standard curve and expressed as copies/gram.

### Histology, immunohistochemistry (IHC) and confocal microscopy

After formalin fixation, tissues were dehydrated, embedded in paraffin, and cut in 5 μm sections. Sections were deparaffinized in xylene (Klinipath, Breda, the Netherlands), rehydrated in descending concentrations of ethanol and pre-treated for optimal antigen retrieval by boiling the slides for 20 min in 10 mM citrate buffer (pH 6.0; Sigma, Zwijndrecht, the Netherlands). Slides were cooled on ice for 30 min and endogenous peroxidase (PO) was blocked with 3% H_2_O_2_ for 15 min at room temperature. For WNV, tissue sections were blocked for 1 h at room temperature with 0.05% Tween20 and 5% rabbit serum in PBS and subsequently incubated overnight with primary goat anti-WNV non-structural protein 3 (NS3) antibody (1:100; R&D Systems, Abingdon, UK) at 4°C. For CHIKV, tissue sections were blocked with 0.05% Tween20 and 5% goat serum in PBS and incubated overnight with rabbit-anti-CHIKV capsid (1:2,500) (van den Doel et al., 2014) at 4°C. After washing, slides were incubated with the secondary antibody [rabbit-anti-goat IgG-PO antibody for WNV, and goat-anti-rabbit IgG-PO for CHIKV (both 1:100, Dako, Eindhoven, The Netherlands)] for 1 h at room temperature and developed with 3-amino-9-ethyl carbazole (AEC, Sigma) substrate, counterstained with Mayer's hematoxylin and mounted with Kaiser's glycerin-gelatin. Screening of sections was done with an Olympus BX51 microscope coupled to a camera (objective 10x and 20x).

### Messenger RNA profiling

Total RNA was isolated from brain samples stored in RNAlater® using Trizol Reagent (Invitrogen, Breda, The Netherlands) and the RNEasy Mini kit (Qiagen, Venlo, The Netherlands). RNA concentrations and OD 260/280 ratios were measured with the NanoDrop ND-1000 UV-VIS spectrophotometer (NanoDrop Technologies, Wilmington, USA). Assessment of RNA quality and purity was performed with the RNA 6000 Nano kit on the Agilent 2100 Bioanalyzer (Agilent Technologies, Palo Alto, CA, USA). RNA (200 ng) was labeled using the MessageAmp Premier RNA Amplification kit (Applied Biosystems) and hybridized to Affymetrix GeneChip® Mouse 4302 Arrays (Affymetrix, ThermoFischer Scientific, Bleiswijk, The Netherlands), according to the manufacturer's recommendations. Image analysis was performed using GeneChip Operating Software (Affymetrix). Microarray Suite version 5.0 software (Affymetrix) was used to generate .dat and .cel files for each experiment. The raw data was deposited in ArrayExpress under access number E-MTAB-5832.

### Microarray processing and analysis

Probe-level data was normalized using quantile normalization and the transformed probe values were summarized into probe set values by the median polish method (Gautier et al., 2004). Probe set wise comparisons between the experimental conditions were performed using limma (Phipson et al., 2016). Correction for multiple testing was achieved by applying a false discovery rate (FDR) of 0.05, calculated using the Benjamini-Hochberg procedure (Benjamini and Hochberg, 1995). Further downstream processing and interpretation of data including principal component analysis (10,000 most variable probesets) and limma analysis (Ritchie et al., 2015) was performed in R version 3.3.1.

### Curation of gene sets

To investigate the different effector pathways of interest, a refined set of genes extensively curated from annotated literature and biological databases was used. Literature containing the keywords: genes, inflammatory molecules in neurodegenerative diseases, pathways and cell death, apoptosis, pyroptosis, necroptosis, autophagy, inflammasome, mitochondrial dysfunction, and neuronal dysfunction, were selected, including both normal and disease states from multiple species. Throughout the annotated literature links to key genes in a particular pathway were also used.

### Gene set and meta-analysis

Gene set analysis based on GOSlim (The Gene Ontology, 2017) and Reactome (Fabregat et al., 2016) pathways was performed using the quantitative self-contained geneset analysis method “fry” from the limma package on probe sets mapped to ensemble mouse identifiers. Results were visualized as heatmaps. Curated gene set analysis and meta-analysis was performed on probe sets mapped to MGI symbol. The curated gene sets were tested for differential expression using the competitive gene set testing approach “camera.” For meta-analysis, the overlap was represented as the Fisher's test odds ratio and 90% confidence intervals. All mappings were performed using Ensembl 87 annotation. When multiple probe sets mapped to genes or identifiers, the probe set with the highest median expression was selected. All analyses were performed in R 3.3.1.

### Quantitative real-time RT-PCR for validations

Results obtained from microarray data for WNV were verified in an experiment using adult mice (WNV: *n* = 8; control: *n* = 6). Expression of 10 genes that were found to be differentially expressed (DE) in the microarray analysis was validated by qRT-PCR. These were as follows, for up-regulated genes: *Gzmb* (Mm00442834_m1), *Ubd* (Mm01972246_s1), *Ccl5* (Mm01302428_m1), *Cxcl9* (Mm00434946_m1), *Tnfaip2* (Mm00447578_m1), *Lcn2* (Mm00809552_s1), and for down-regulated genes: *Mfge8* (Mm00500549_m1), *Slc7a10* (Mm00502045_m1), *Aplnr* (Mm00442191_s1), and *Map6* (Mm00447898_m1). The expression of three pyroptosis markers (as indicated below) were also validated in the WNV-infected adult mice. *Gapdh* (mM99999915_g1) was used as the endogenous reference gene for normalization of target gene expression.

Results obtained from microarray data for CHIKV were verified with qRT-PCR using the extracted mRNA obtained for transcriptomic analysis for a selection of six genes involved in apoptosis and pyroptosis. The following markers were measured: *caspase-1* (Mm00438023_m1), *caspase-3* (Mm01195084_m1), *caspase-9* (Mm00516563_m1), *IL-18* (Mm00434225_m1), *IL-1*β (Mm01336189_m1), and *TNF-*α (Mm00443259_g1). The house-keeping gene β*-actin* (ACTB 4351315) was used as the endogenous reference gene for normalization of target gene expression. A house-keeping gene was selected when the *C*_*T*_-values between the control and infected groups did not vary.

Purified RNA was converted to cDNA using Superscript III and oligo dT primer (Invitrogen) according to the instructions of the manufacturer. Expression of the different target genes was determined using TaqMan® Universal PCR Master Mix and TaqMan Gene Expression Assay primer and probe (FAM-MGB) sets (Applied Biosystems) on a 7500 Fast Real-Time PCR System (Applied Biosystems) under the following conditions: activation at 50°C for 2 min followed by 95°C for 10 min, and 40 cycles of denaturing at 95°C for 15 s and annealing at 60°C for 1 min. Relative expression fold-change was calculated by the 2^−ΔCt^ method and for WNV validations presented as the expression ratio of the mean *C*_T_ of WNV-infected over mock-infected mice. Gene expression values were analyzed with a two-tailed, non-parametric Mann-Whitney test (Graph Pad Prism version 6). Values of *P* ≤ 0.05 were considered statistically significant.

## Results

### Different kinetics of WNV and CHIKV infections in young mice

**Figure 1:**
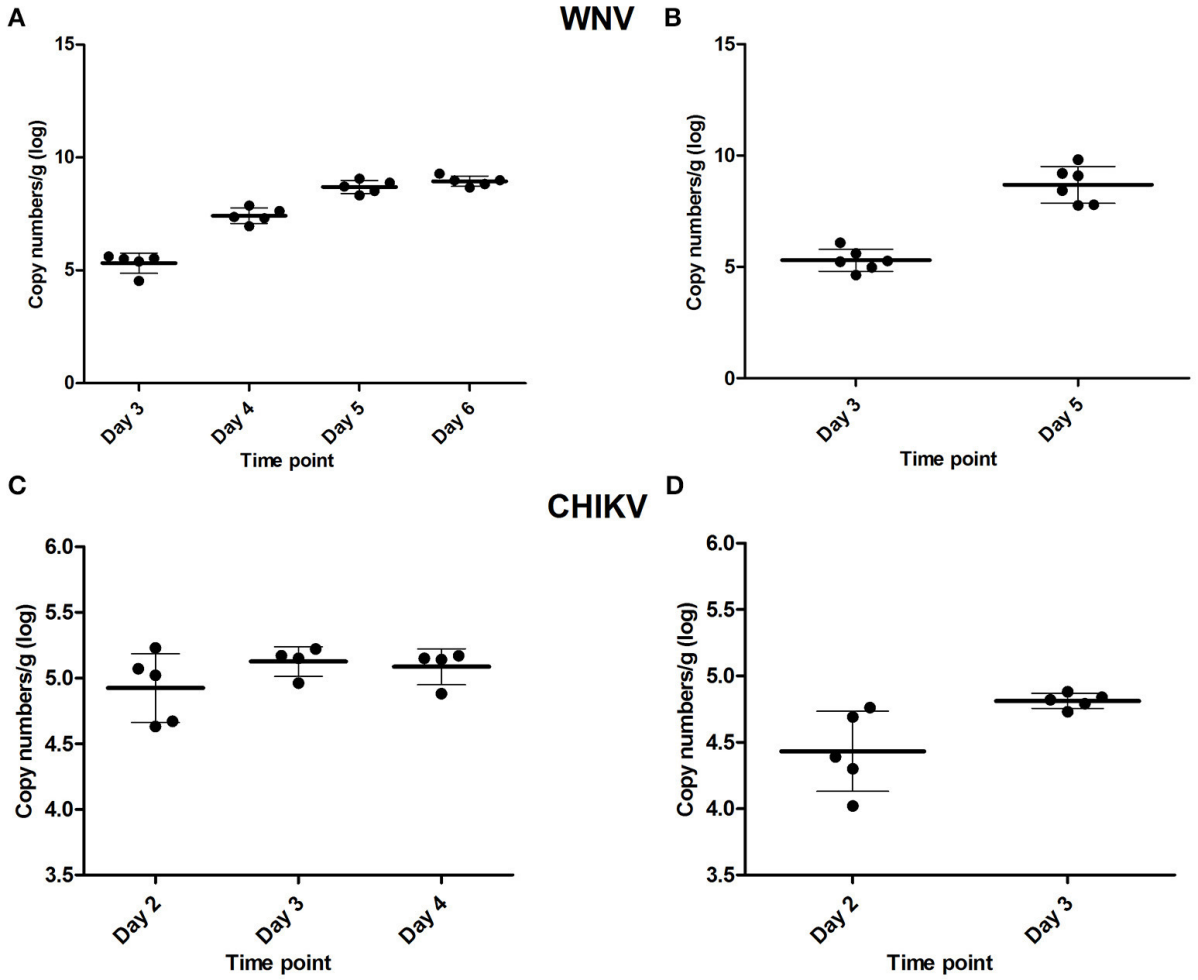
Viral RNA titers found in the brain of WNV-infected mice determined during the **(A)** kinetics experiment, and **(B)** microarray experiment, as well as viral RNA titers found in the brain of CHIKV-infected mice of the kinetics experiment **(C)**, and in the cerebellum of mice used for the microarray **(D)**. Viral titers are given as copy numbers per gram of brain; error bars represent the standard deviation.

To determine the time point at which virus was first detected in the brain after peripheral inoculation (i.e., the early time-point) and the onset of neuroinvasive disease (i.e., the late time-point), a kinetics experiment was performed in young mice. For WNV, mice were asymptomatic up to and including day 3 p.i. The first signs of illness, such as lethargy, decreased mobility, and balancing problems, appeared on day 4 p.i., and by day 5 and 6 these symptoms had progressed to moribund state and/or hind-leg paralysis. Infection of the brain was confirmed at all the time points tested (Figure 1A) using qRT-PCR. On day 3, ~10^5.4^ RNA copies were present in the brain, which increased to ~10^7.4^ on day 4, 10^8.7^ on day 5 and 10^9^ RNA copies by day 6. Based on the clinical presentation and viral RNA titers in the brain, day 3 was chosen as the early time point (WNV-E) of infection and day 5 as the late time point (WNV-L), at which the majority of the animals had reached the humane end-point of the experiment. All mice euthanized at the early and late time point in the subsequent experiment were positive for virus in the brain with mean titers of ~10^5.6^ and 10^9.2^ RNA copies, respectively (Figure 1B). Mice of the control group were negative for virus in the brain as expected.

For CHIKV, infected mice sacrificed on day 2 did not yet display any clinical signs, while CNS involvement (tetanus-like phenotype) was observed on day 3 in the majority of the animals. Viral RNA was detected in brains collected on days 2, 3, and 4 (Figure 1C). Day 2 was selected as the early time point of infection (CHIKV-E), while day 3, at which tetanus-like manifestations (characterized by an inverse tetanus-like posture) were observed, was considered as the late time point (CHIKV-L). To determine if viral RNA was present in the brains of infected mice, the cerebellum was tested and viral RNA was detected in all mice euthanized on day 2 and 3 (Figure 1D).

### Both WNV and CHIKV infect neurons in brains of young mice

**Figure 2:**
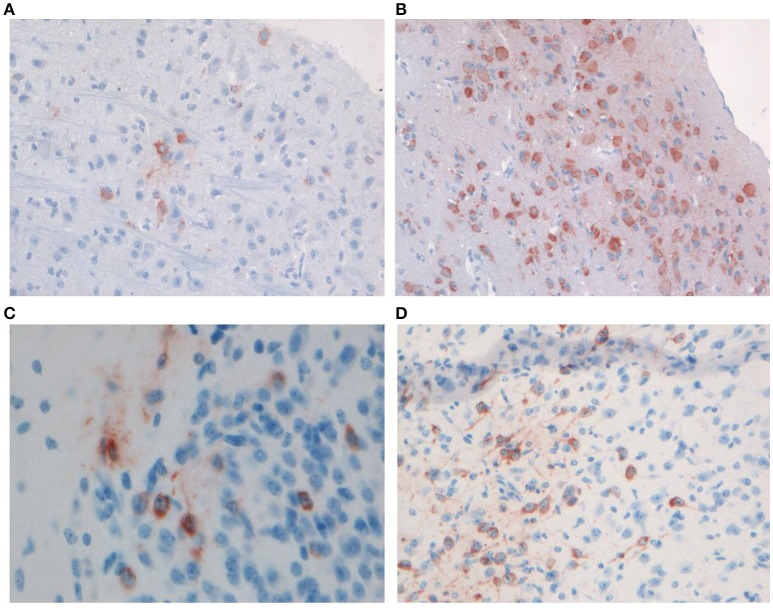
Representative staining of neurons with anti-WNV-NS3 in the brains of C57BL/6 mice euthanized on **(A)** day 3 (magnification 20×) and **(B)** day 5 (magnification 20×) of the kinetics experiment, and staining of neurons with anti-CHIKV-capsid in the midbrain of C57BL/6 mice euthanized on **(C)** day 2 (magnification 40×) and **(D)** day 4 (magnification 20×) of the kinetics experiment.

Productive infection with WNV- and CHIKV was confirmed by IHC staining using brain samples of the mice from the kinetics experiment. A low amount of WNV-infected neurons was demonstrated in the brains of mice at the early time point (Figure 2A), whereas more extensive staining of neurons across the brain was found at the late time point (Figure 2B), in line with the qRT-PCR results.

IHC staining for CHIKV viral antigen revealed few positive neurons in the brains of mice sacrificed on day 2 p.i. (Figure 2C), while by day 3 p.i. the virus had spread extensively to other areas of the brain (Figure 2D).

### Differences in differentially expressed genes between the early and late time points of infection and between WNV and CHIKV infected young mice

**Figure 3:**
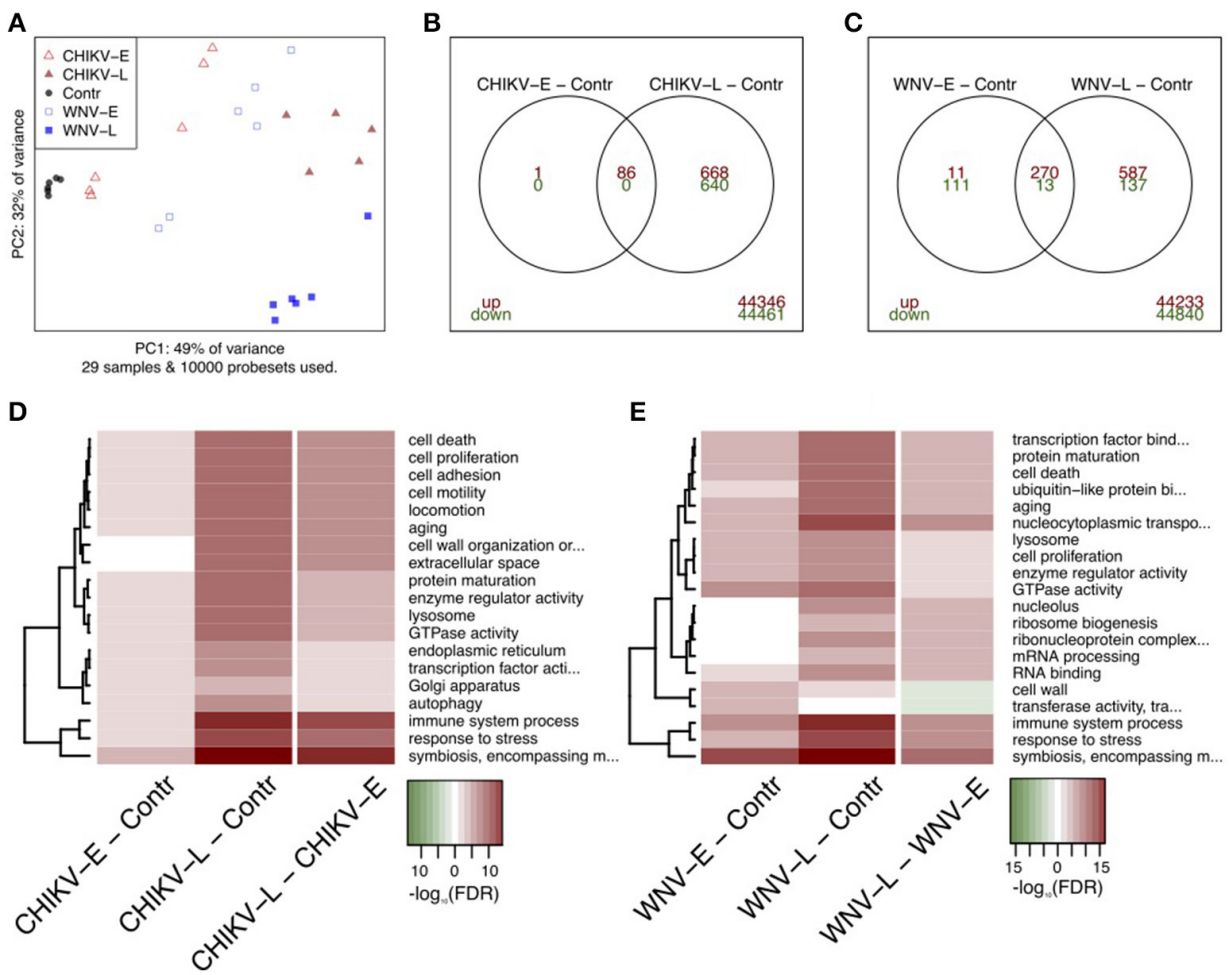
Analysis of transcriptome profiles of brain tissue after infection with CHIKV and WNV. **(A)** PCA analysis showing the different infected groups and controls. **(B,C)** Differentially expressed probe sets for early and late during CHIKV infection, and early and late during WNV infection, respectively. Up-regulated genes had a fold change of ≥2 and a FDR of <0.05 shown as up (red) or down-regulated (green). **(D,E)** Quantitative gene set analysis using GOslim annotation for the indicated contrasts. Up-regulated gene sets are indicated in red, down in green. The top-10 gene sets for every contrast are included in the heatmaps.

To obtain an overall view of the effect of WNV and CHIKV infection on the brain at the mRNA-level, we applied the principal component analysis (PCA) to evaluate the transcriptome profiles obtained at both early and late time points (Figure 3A). For this analysis, the 10,000 most variable probe sets were used and the results were depicted as a two-dimensional plot. The first component, which explains 49% of the variability in these probe sets, largely concurred with the time since inoculation or progression of infection as it aligned all the time points from control to 5 days p.i. The second principal component (32% of variability) primarily separated the WNV-L samples from the other groups. Taken together, PCA transcriptome analysis is in line with the observation that the early and late groups displayed a distinct clinical phenotype.

We compared the host transcriptional signatures in samples collected early (CHIKV: day 2; WNV: day 3) and late (CHIKV: day 3; WNV: day 5) during the infection process (Figures 3B,C). In general, a larger number of genes were differentially expressed (DE) as the infection progressed, both in WNV and in CHIKV. By applying a fold change of ≥2 and a FDR of <5%, we identified 87 and 1395 probe sets in CHIKV-E and CHIKV-L when compared to the control, respectively (Figure 3B). The CHIKV-E set was mostly a subset of the larger CHIKV-L signature. For WNV, we identified 405 and 1007 DE probe sets when comparing WNV-E and WNV-L to the control, respectively. The majority of these probe sets were up-regulated and there was clear overlap between the early and late signatures, but less than in CHIKV (Figure 3C).

For the late vs. early comparisons, 390 genes were DE for WNV and 310 genes for CHIKV, of which 199 genes were uniquely DE for WNV and 119 for CHIKV (**Figure 6B**). The top 10 most DE transcripts within the microarray analysis were selected based on expression value (log ratio) and are summarized in Tables 1, 2. Of the top 10 genes, five up-regulated (*Lcn2, Cxcl9, Tnfaip2, Ccl5*, and *Lilrb4*) and one down-regulated gene (*Slc40a1*) were found to be common to both the WNV and CHIKV late vs. early comparisons.

**Table 1:** Top 10 most up-regulated genes as determined by log_2_ fold change in the brains of WNV- and CHIKV-infected mice for the late to early stage comparison.

**WNV-L vs**. **E**				**CHIKV-L vs**. **E**			
**Symbol**	**Entrez gene name**	**Log_2_ fold change**	***P*-value**	**Symbol**	**Entrez gene name**	**Log_2_ fold change**	***P*-value**
Lcn2	Lipocalin 2	4.87	6.81E-10	Cxcl10	Chemokine (C-X-C motif) ligand 10	5.09	4.38E-13
Cxcl9	Chemokine (C-X-C motif) ligand 9	4.80	4.46E-11	Lcn2	Lipocalin 2	4.73	4.48E-09
Ubd	Ubiquitin D	4.76	2.59E-17	Ccl5	C-C motif chemokine ligand 5	4.19	4.22E-10
Tnfaip2	TNF alpha induced protein 2	4.71	4.00E-13	Ifi204	Interferon activated gene 204	4.18	5.19E-11
Ccl5	C-C motif chemokine ligand 5	4.43	3.01E-11	Gbp2	Guanylate binding protein 2	4.13	1.34E-13
Gzmb	Granzyme B	4.39	4.20E-15	Lilrb4	Leukocyte immunoglobulin like receptor 4	4.12	5.84E-11
Slfn12l	Schlafen family member 12 like	4.07	1.39E-08	Acod1	Aconitate decarboxylase 1	4.08	6.77E-12
Ifnb1	Interferon beta 1	3.89	9.13E-16	Cxcl9	Chemokine (C-X-C motif) ligand 9	4.02	6.94E-09
Ccl13	C-C motif chemokine ligand 13	3.89	1.92E-13	Ms4a4b	Membrane-spanning 4-domains, subfamily A, member 4B	3.99	3.74E-08
Lilrb4	Leukocyte immunoglobulin like receptor B4	3.89	6.82E-11	Tnfaip2	TNF alpha induced protein 2	3.89	1.48E-10

**Table 2:** Top 10 most down-regulated genes as determined by log_2_ fold change in the brains of WNV- and CHIKV-infected mice for the late to early stage comparison.

**WNV-L vs**. **E**				**CHIKV-L vs**. **E**			
**Symbol**	**Entrez gene name**	**Log_2_ fold change**	***P*-value**	**Symbol**	**Entrez gene name**	**Log_2_ fold change**	***P*-value**
Aplnr	Apelin receptor	−2.46	1.04E-11	Gpr34	G-protein coupled receptor 34	−1.68	2.65E-08
Slc7a10	Solute carrier family 7 member 10	−2.26	1.56E-13	Slc40a1	Solute carrier family 40 member 1	−1.51	2.57E-12
Hbb-b2	Hemoglobin, beta adult minor chain	−2.16	2.57E-09	Ednrb	Endothelin receptor type B	−1.48	7.32E-07
Hba1/Hba2	Hemoglobin subunit alpha 2	−2.09	3.76E-07	Idi1	Isopentenyl-diphosphate delta isomerase 1	−1.38	7.99E-05
Ca8	Carbonic anhydrase 8	−1.83	2.31E-07	Meg3	Maternally expressed 3	−1.35	0.0037
Nrarp	NOTCH-regulated ankyrin repeat protein	−1.77	3.03E-10	Gls	Glutaminase	−1.31	6.63E-06
Slc40a1	Solute carrier family 40 member 1	−1.74	4.29E-12	Zfp826	Zinc finger protein 826	−1.31	0.0022
Hes5	hes family bHLH transcription factor 5	−1.71	6.74E-11	P2ry12	Purinergic receptor P2Y12	−1.26	1.11E-09
Mfge8	Milk fat globule-EGF factor 8 protein	−1.66	6.51E-13	Ttll1	Tubulin tyrosine ligase like 1	−1.25	5.66E-07
Map6	Microtubule associated protein 6	−1.62	5.47E-12	Hspa4l	Heat shock protein family A (Hsp70) member 4 like	−1.25	0.0048

Subsequently, the biological processes reflected by the up-regulated genes were identified (Figures 3D,E). The top DE pathways for both CHIKV and WNV included up-regulation of “immune system processes,” “response to stress,” and “symbiosis, encompassing mutualism through parasitism,” which is a generic gene set that also includes host-virus interactions. Other notable DE gene sets included “cell proliferation,” “aging,” and “cell death processes,” in particular at the late time points in both CHIKV and WNV infection. When we compared late to early transcriptome profiles, we observed the same processes to be up-regulated, which shows that the infection progressed after day 3 p.i. (Figures 3D,E). In the comparison of the late-stage WNV and CHIKV infection with the early-stage, the most significant (in terms of *P*-value) gene ontology terms associated with up-regulated genes included: “autophagy,” “immune system process,” “transcription factor activity,” and “response to stress.” A full list of GOslim terms associated with DE genes is shown in Table S1 in the Supplemental Material.

Although late-stage CHIKV infection resulted in a tetanus-like phenotype distinct from WNV neuroinvasive disease, gene set analysis of CHIKV showed that a set of pathways similar to WNV is expressed at the late stage of disease, including up-regulation of “response to stress,” “cell proliferation,” “immune system processes,” and “lysosome process.”

### Differential gene expression is associated with immune response pathways in WNV and CHIKV infected young mice

To further characterize the immune processes present in mice with neuroinvasive disease caused by infection with WNV and CHIKV (late vs. early), we used immune pathways from REACTOME to find pathways and processes that were altered upon viral infection. Many of the highly expressed transcripts were involved in innate and adaptive immunity. For instance, the activation of the inflammatory response is dependent on transcription factors such as NFκB, USF1, SP1, and FOX (i.e., FOXJ1, FOXN1, FOXO1, FOXP3), of which five were up-regulated in the WNV-infected animals and seven in the CHIKV-infected animals (late vs. early; Table S2). Many pathways typically associated with viral infection were up-regulated early and equally upon CHIKV and WNV infection, including interferons, class I antigen presentation and complement pathways. Pathogen-recognition receptors (PRRs) such as toll-like receptors were up-regulated more strongly in WNV than in CHIKV infection, both at early and late time points (Figure S1). Generally speaking, similar pathways were found to be DE in both WNV and CHIKV infections.

**Figure 4:**
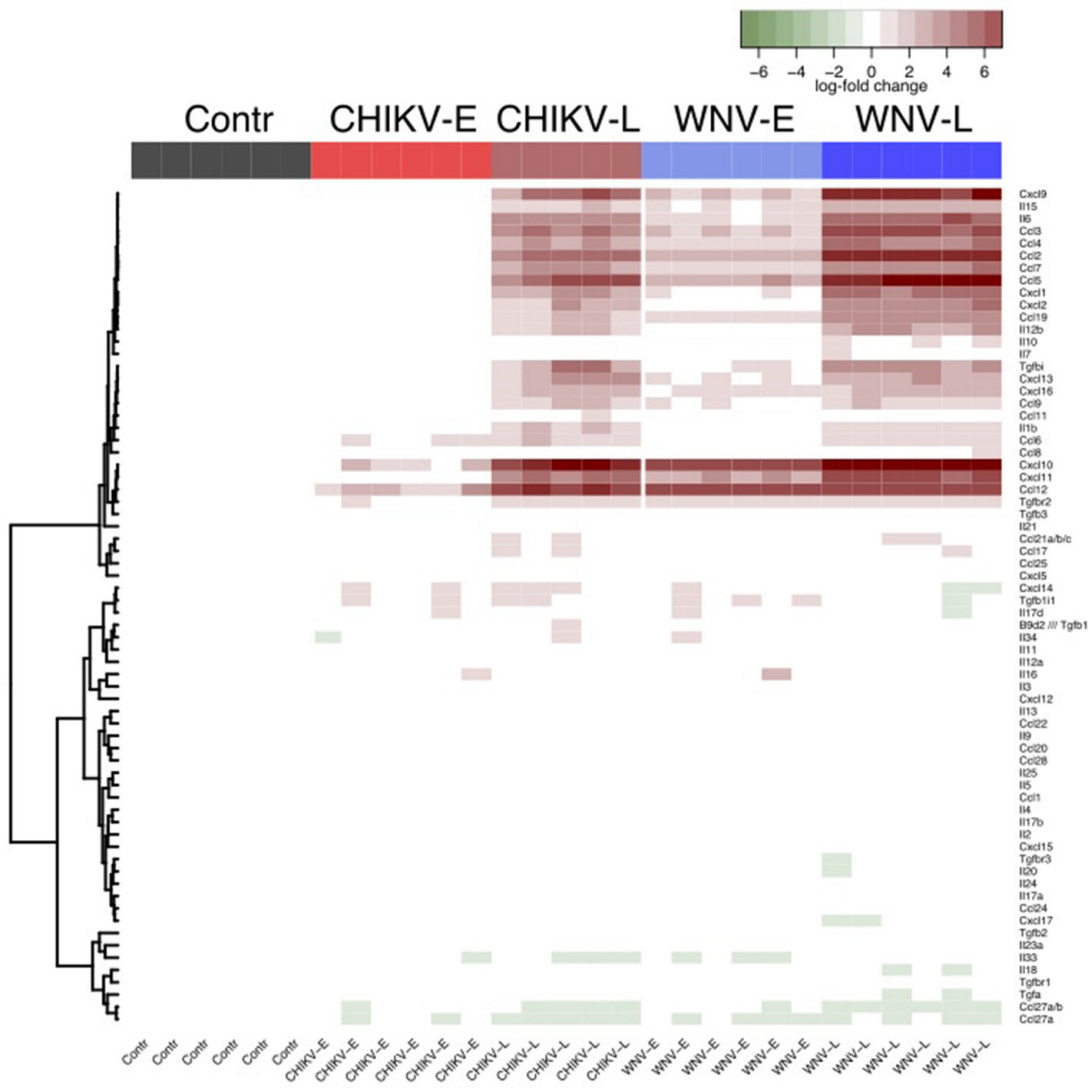
Heatmap to show the relative enrichment of Reactome immune gene sets following a self-contained gene set test. Cytokine and chemokine expressions are given relative to the median of the control samples.

To better examine differences in inflammatory mediators known to play a role in neurodegenerative diseases, we looked closely at specific cytokines and chemokines and plotted their fold changes relative to the control (Figure 4), and also compared late vs. early (Tables S2, S4). Differential regulation of cytokines and chemokines was observed between CHIKV and WNV, both at the early and late time points. Fold changes tended to be slightly higher in WNV infection compared to CHIKV, but did typically involve the same mediators. Briefly, 7 of 12 genes encoding cytokines (Table S2) and 16 of 21 genes encoding chemokines (Table S4) were differentially regulated in WNV-L (vs. early), while 8 cytokine genes and 14 chemokine genes were differentially regulated for CHIKV-L (Tables S1, S3). Inflammatory molecules such as IL-6, IFN-γ, CCL-2, -3, -4, -5, CXCL10, and CXCL2 were up-regulated in both WNV-L and CHIKV-L (Figure 4, Tables S1, S3). In addition, the gene expression of molecules that regulate the immune response, such as TNFRSF1A, CXCR4, CD47, and MAPK12 was up-regulated during WNV-L and CHIKV-L (compared to their early time point) (Table S3).

### Different cell death pathways are activated in young mice with neuroinvasive disease

**Figure 5:**
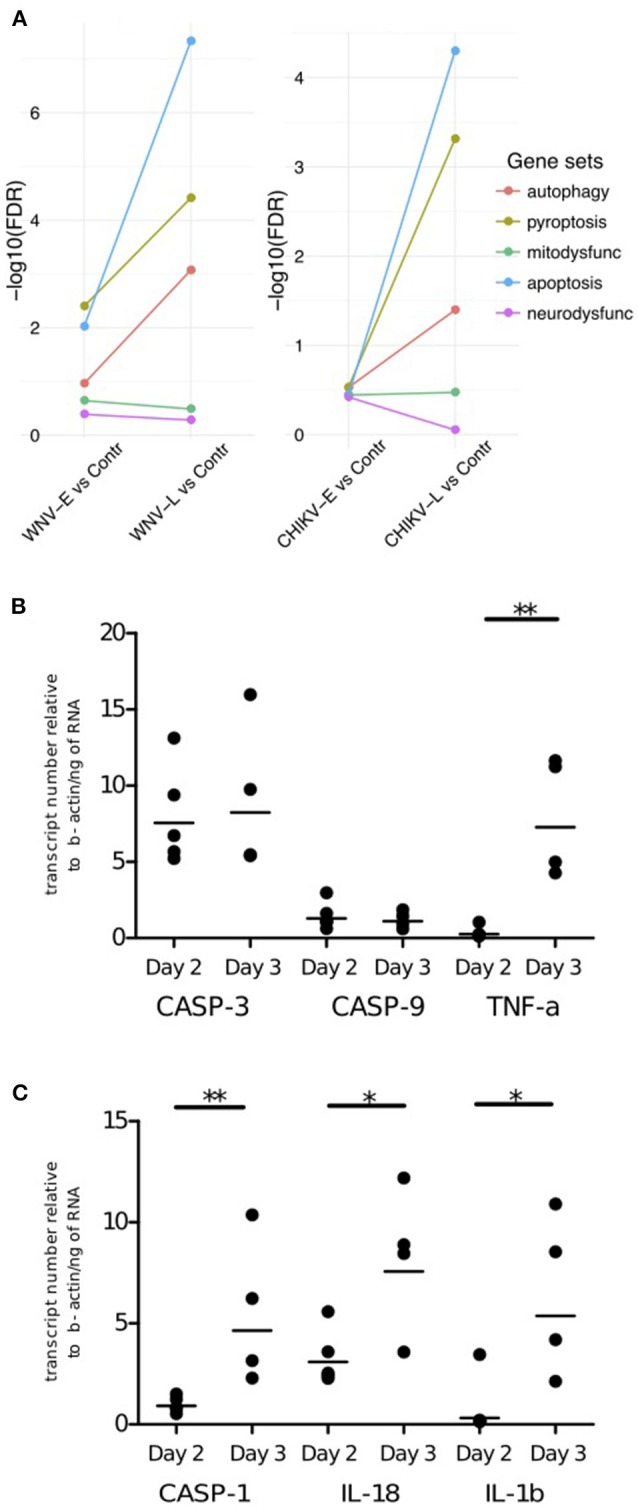
Cell death pathways investigated in WNV- and CHIKV-infected mice. **(A)** Competitive gene set test results for curated gene sets of cell death pathways found in WNV (left) and CHIKV (right) neuroinvasive disease. The genes included in the genesets are provided in the supplementary material. qRT-PCR validations of **(B)** apoptosis and **(C)** pyroptosis microarray data for CHIKV. qRT-PCR was performed on bulk brain (cerebrum and cerebellum) of CHIKV-S27 infected 9-day-old C57BL/6 mice euthanized on day 2 (*n* = 5) and day 3 (*n* = 4) p.i. to assess caspase-3 (casp-3) and -9(casp-9) and TNF-α expression levels (apoptosis) and caspase-1 (casp-1), IL-18, and IL-1β (pyroptosis) expression levels. Mouse β-actin (Actb) was used as the housekeeping gene, and all data were expressed compared to Actb. Each point in the graph represents the expression value for the corresponding apoptosis marker of 1 sample. The mean is represented here as a black line. ^*^P ≤ 0.05, ^**^P ≤ 0.01: statistically different (two-tailed, Mann-Whitney test).

The pathogenesis of neuroinvasive disease is believed to be the result of cell death but may also involve cell dysfunction. Inflammation is known to be associated with dysfunction and cell death, which we also observed in the generic gene set analysis as the “cell death” pathways and many genes associated with neurological disease were up-regulated (Figures 3D,E). To pinpoint which of these processes are most prominent in WNV and CHIKV infection of the brain and elucidate the involvement of cellular dysfunction, we compared mitochondrial and neuronal dysfunction curated gene sets (Tables S5, S6) to cell death-related curated gene sets including apoptosis (Table S7), pyroptosis (Table S8), and autophagy (Table S9). By using a competitive gene set testing approach, we were able to establish the gene sets that were most up-regulated relative to the others (Figure 5A). The analysis indicated that neuronal and mitochondrial dysfunction processes had less impact on the transcriptome than the cell death pathways. Conversely, the cell death pathways pyroptosis and apoptosis were highly enriched at the late stage (WNV-L and CHIKV-L) when compared to the early stage. Of the cell death pathways, apoptosis had the strongest signature in both CHIKV-L and WNV-L compared to early (Figure 5A).

Given the small set of genes currently known to be involved in necroptosis, a competitive gene set testing approach could not be carried out. However, four of the six genes important for promoting necroptosis were found up-regulated in both WNV and CHIKV neuroinvasive disease (WNV-L or CHIKV-L vs. Ctrl; Table 3), of which three form the necroptosome (MLKL, RIPK1, and RIPK3), a supra-molecular structure required for further transduction of the necroptosis signal into mitochondria. Up-regulation of components of this pathway was found to progress from the early to the late stage in both viral infections. Concurrent with the up-regulation of this pathway through disease progression was also the up-regulation of inhibitors of necroptosis, such as BIRC2 and BIRC3, indicating that the pathway was most likely activated (Table 3).

**Table 3:** Differential regulation of genes involved in necroptosis in WNV- and CHIKV-infected mice (log2fold change).

	**Necroptosis**	**WNV-E vs. Ctrl**	**WNV-L vs. Ctrl**	**WNV-L vs. WNV-E**	**CHIKV-E vs. Ctrl**	**CHIKV-L vs. Ctrl**	**CHIKV-L vs. CHIKV-E**
**Symbol**	**Entrez gene name**	**Log**_2_ **fold change**	**Log**_2_ **fold change**	**Log**_2_ **fold change**	**Log**_2_ **fold change**	**Log**_2_ **fold change**	**Log**_2_ **fold change**
CYLD	CYLD lysine 63 deubiquitinase	0	0	0	0	−0.90	0
MLKL	Mixed lineage kinase domain like pseudokinase	1.37	2.47	0	0	1.98	1.63
RIP1 (RIPK1)	Receptor interacting serine/threonine kinase 1	0.19	1.22	1.00	0	0.62	0.69
RIP3 (RIPK3)	Receptor interacting serine/threonine kinase 3	0	0.52	0.46	0	0.26	0.28
CFLAR (C-FLIP)	CASP8 and FADD like apoptosis regulator	0.38	1.78	1.40	0	1.38	1.43
FADD	Fas associated via death domain	0	0	0	0	0	0
BIRC2 (cIAP1)	Baculoviral IAP repeat containing 2	0	0.97	1.09	0	0.30	0.44
BIRC3 (cIAP2)	Baculoviral IAP repeat containing 3	0.59	2.64	2.05	0	1.69	1.65

### Validation of microarray data by qRT-PCR

The gene expression of an almost equal selection of the top 10 most highly up- or down-regulated genes observed in young mice infected with WNV, and deemed most relevant for viral infection, immunology or for expression in the brain, was validated by qRT-PCR by using brains of adult mice infected with WNV and euthanized at the severe stage of infection, and compared to the brains of mock-infected mice. Out of the 10 validated genes, all genes showed a similar direction of differential expression as compared to the microarray, and therefore deemed as genes relevant to WNV infection (Table 4).

**Table 4:** Comparison of the gene expression of a selection of the top 10 differentially regulated genes with qRT-PCR data obtained from adult mice infected with WNV.

**Gene symbol**	**Log**_**2**_ **fold change**	
	**Microarray analysis**	**qRT-PCR**
	**WNV-L vs. Ctrl**	**WNV-L vs. Ctrl**
Cxcl9	6.33	7.81
Ubd	5.21	3.41
Gzmb	4.89	7.08
Ccl5	6.23	7.81
Tnfaip2	5.82	2.93
Lcn2	6.12	4.17
Mfge8	−1.36	−2.27
Slc7a10	−2.02	−0.99
Aplnr	−1.82	−1.01
Map6	−1.15	−1.65

*Expression was normalized according to the house keeping gene GADPH by using the 2^−ΔCt^ method and are presented as the expression ratio of the mean C_T_ of WNV-infected over mock-infected mice*.

In addition, as the role of apoptosis in the pathogenesis of WNV has already been intensely investigated in the literature, we only validated the differential regulation of the pyroptosis markers in adult mice. In accordance with the microarray data, two of the three markers, caspase-1 and IL-1β were found to be upregulated by a log_2_ fold change of 3.21 and 1.64, respectively (data not shown), suggesting potential involvement of this pathway in adult mice with severe WNV disease as well.

As CHIKV is not able to cause neuroinvasive disease in (adult) immunocompetent mice, validations were conducted on the same samples as the microarray analysis rather than in another mouse model. Analyzing the expression of three genes involved in apoptosis (*caspase-3* and *-9* and *TNF-*α; Figure 5B) and three genes involved in pyroptosis (*caspase-1, IL-18* and *IL-1*β; Figure 5C), an increase over time in TNF-α mRNA levels (*P* = 0.02) was observed, whereas the mRNA level of caspase-3 and -9 did not show any significant increase or decrease over time. An increase in mRNA over time was also observed for caspase-1 (*P* = 0.02), IL-18 (≥ 0.05, not significant), and IL-1β (*P* = 0.03). This supports the potential involvement of pyroptosis in CHIKV neuroinvasive disease.

### Overlap in transcriptome signatures of neuroinvasive disease in mouse brains after viral infection

To determine the similarity of the transcriptomic profiles of WNV and CHIKV neuroinvasive disease in our study to other viral models of neuroinvasive disease, the up- and down-regulated genes in the brains of WNV and CHIKV infected mice obtained in our study were compared to each other and to transcriptome data from WNV, Japanese encephalitis virus (JEV) and reovirus infections in the brain from the literature (Clarke et al., 2014a; Kumar et al., 2016). To this end, all genes were mapped to MGI symbols to allow for cross-referencing between datasets. Comparing transcriptome profiles between different studies is challenging given the diversity in results that presumably result from experimental and technical differences between studies. Furthermore, any difference in kinetics between datasets will also contribute to differential gene expression between viruses. As a result, we focused on overlap within studies, rather than between studies.

**Figure 6:**
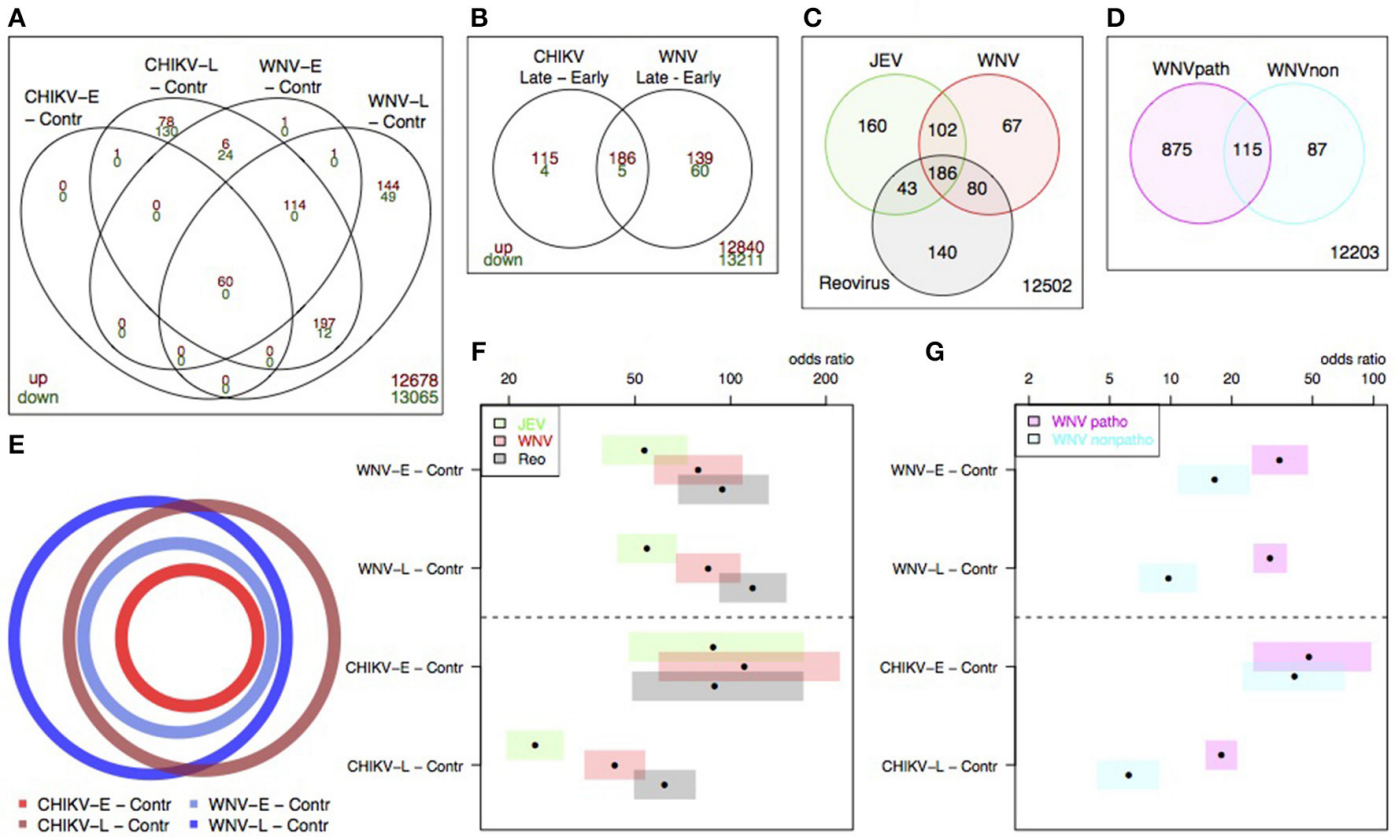
Overlap in differential expression signatures within datasets for our data **(A,B,C)** and Clarke et al. **(C)** and Kumar et al. **(D)**. The overlap between our CHIKV and WNV data is stated in **(A)**, and schematically depicted in **(E)**. The overlap between our data and the Clarke et al. **(F)** and Kumar et al. sets **(G)** is given by the Fisher's odds ratio and confidence intervals (bars).

When we compared the transcriptional profiles of WNV to those of CHIKV, we observed genes that were specific to either WNV or CHIKV, but we also saw a large overlap between differentially expressed genes (Figures 6A,B). Using a euler diagram to depict the gene expression profiles from Figure 6A (Figure 6E), we observed that the early time points were practically subsets of the later time point gene lists, demonstrating progressive infection for both viruses. Additionally, the overlap between WNV and CHIKV was large enough to conclude that in our experiments, the pathogenesis of WNV and CHIKV in the brain at mRNA level was largely similar (Figures 6A,B,E).

To further compare our results to other published studies, we obtained the differentially expressed gene lists from the Clarke et al. and Kumar et al. studies and mapped them to unique MGI (Figures 6C,D). Using the Fisher's test odds ratio and 95% confidence intervals to enumerate the overlap between the sets of DE genes, we evaluated the overlap in the response to different virus infections. When the overlap between our results for WNV and the Clarke et al. sets was compared (Figures 6C,F), reovirus tended to have the largest overlap with our WNV signatures, for both early and late, while the overlap between our and the Clarke et al. WNV data was slightly smaller at both the early and late time point (Figure 6F).

For CHIKV-E, no difference in overlap between the Clarke et al. sets was observed at the early time point. At the late time point, the overlap with Clarke et al. is smaller, but the overlap with reovirus was the largest. Furthermore, our WNV signature exhibited a larger overlap with the pathogenic WNV strain rather than with the attenuated strain used by Kumar et al (Figure 6G). As with the Clarke et al. overlap, the overlap with CHIKV-E was larger than the overlap with CHIKV-L.

Taken together, the meta-analysis shows that our WNV results tended to have consistently high overlaps with the signature sets from the selected literature studies, but that CHIKV induced expression of a slightly different set of genes at the late time point that limited its overlap with other sets.

## Discussion

WNV has had an increasing public health impact since its emergence in the Western Hemisphere in 1999. Also the emergence of CHIKV in the Pacific and the Americas had a tremendous impact on the affected populations. Both viruses have caused neuroinvasive disease in high-risk patients, and despite the progress in understanding the mechanisms of neuroinvasive disease, the precise pathways that are active during the late stage of WNV and CHIKV severe disease are not well-established. In this study, we used transcriptomics to identify the effector pathways operating during the late stage of WNV and CHIKV neuroinvasive disease and we showed that several cell death pathways are simultaneously activated in mice with severe disease.

From a conceptual point of view, the pathogenesis of WNV and CHIKV infection can be considered a branching cascade where the virus simultaneously initiates multiple pathological pathways that then collectively contribute to neuronal death and dysfunction. For most triggers of neurologic disease, which consist of either proteinopathy or infection, multiple hypotheses exist regarding the mechanism(s) of pathogenesis, including neuronal dysfunction and/or cell death. It has been shown that common effector pathways are triggered across multiple neurological (inflammatory) diseases, such as inflammation (Glass et al., 2010; Amor et al., 2014), apoptosis (Honig and Rosenberg, 2000; Mattson, 2000), proteasome dysfunction (Dantuma and Bott, 2014), stress responses (Kim et al., 2015; Scheper and Hoozemans, 2015), mitochondrial dysfunction (de Castro et al., 2010), axonal transport deficits (Millecamps and Julien, 2013), neuronal cell cycle induction (Wang et al., 2009), pyroptosis (Guo et al., 2015), autophagy (Ghavami et al., 2014), and necrosis (Troulinaki and Tavernarakis, 2012). We sought to determine whether pathways of programmed cell death and dysfunction were activated during the late stage disease of WNV and CHIKV infection.

To study and compare the response in the brain to infection by these two viruses, we used nine-day old C57BL/6 mice in which both WNV and CHIKV are able to cause neuroinvasive disease. In order to identify the effector pathways differentially expressed during severe disease it was important to compare the late stage of disease with an early time point at which virus was already present in the brain but mice were not yet showing signs of neurologic disease. However, one caveat in the comparison of transcripts between these two infection models is the difference in the replication kinetics of the respective viruses, as a valid comparison can only be built if the kinetics of the viruses and corresponding host responses are nearly identical. CHIKV replicates faster *in vitro* than WNV (unpublished observations), which was “confirmed” in this study by the fact that CHIKV entered the brain of infected mice earlier and caused disease more rapidly than WNV. One way in which we could have circumvented the different replication kinetics and pathogenic mechanisms in the periphery and thereby improve our evaluation of WNV- and CHIKV-induced gene expression in the brain would have been by using intracranial inoculation. Other experimental differences in our study between the two virus models included viral inoculation dose and the virus titers found in the brains of the mice. As a result, we only compared the effector pathways observed in the animals at the time that they were experiencing signs of severe neuroinvasive disease.

Furthermore, the bulk brain was used to analyze the transcriptomics of WNV- and CHIKV-infected mice. Although the use of bulk brain may have reduced the resolution of the assay and subsequently decreased the sensitivity for the identification of effector pathways, we reasoned that infection of the brain with both viruses would be extensive and still allow for identification of prominent pathways in the late stage of infection. Even though we have used quantitative gene set analysis techniques that are highly sensitive, we may have missed identification of certain pathways that were close to the background noise. Furthermore, it is safe to assume that the (curated) pathways and gene sets that we tested did not include all the relevant molecules, even though the quality and quantity of gene sets is increasing. Moreover, certain pathways make use of proenzymes or immature proteins of which their activation cannot be determined by gene expression.

Neuroinflammation and cell death have been shown to occur in both infection and neurodegenerative diseases, indicating that common effector pathways may be involved in neurologic diseases. The mechanisms of neuronal injury following WNV and CHIKV infection still remain incompletely characterized but may be caused by direct cytolytic effect of the virus, programmed cell death or immunopathology (Gosztonyi, 2001; Koyuncu et al., 2013). In our study, we observed upregulation of apoptosis markers in both WNV and CHIKV infected mice, and our results are consistent with the transcriptome studies performed in the brains of mice infected with WNV that show expression of apoptosis markers such as caspase-4, -7, and -8 (Bourgeois et al., 2011; Clarke et al., 2014a; Kumar et al., 2016). Furthermore, infection of a range of cells *in vitro* as well as infection of mice with other flaviviruses, such as St Louis encephalitis virus, Murray Valley encephalitis virus and JEV, have also been shown to induce apoptosis (Matthews et al., 2000; Parquet et al., 2002; Myint et al., 2014), suggesting a central role for apoptosis in the pathogenesis of viral encephalitis.

Furthermore, in our study we also observed differential expression of the autophagy pathway in both infection models. Autophagy is believed to be an important survival pathway activated in response to infection; however, studies exist that suggest either a protective (Joubert et al., 2012; Judith et al., 2013; Kobayashi et al., 2014) or pathogenic role (Krejbich-Trotot et al., 2011b) for autophagy during infection with WNV and CHIKV, as well as limited involvement (Beatman et al., 2012; Vandergaast and Fredericksen, 2012).

Despite intensive investigation of apoptosis, little research has been done to understand the role of other cell death pathways in the pathogenesis of WNV and CHIKV encephalitis. In this study, we found evidence for pyroptosis involvement during the later stage of disease for both WNV- and CHIKV-infected mice, which was validated in adult mice infected with WNV. Pyroptosis is an inflammatory form of programmed cell death that also takes place in the CNS and involves the formation of the pyroptosome or inflammasome, activation of caspase-1 and the subsequent release of the proinflammatory cytokines IL-1ß and IL-18 (Chen et al., 2016). Caspase-1 and IL-18 have also been shown to be produced in neurodegenerative diseases such as Alzheimer's disease and Multiple sclerosis (Losy and Niezgoda, 2001; Huang et al., 2004; Sutinen et al., 2012; Saco et al., 2014). Evidence for pyroptosis has also been found in viral diseases, such as dengue (Wu et al., 2013) and adenovirus (Barlan et al., 2011) infections. Molecules important for forming the inflammasome, such as NLRP3 and NLRC5 have been found elevated in mouse brains infected with WNV-NY99 (Kumar et al., 2016). In our study, these molecules, in addition to the other aforementioned ones, were investigated and found to be up-regulated in WNV and CHIKV severe disease (data not shown), providing stronger evidence for the involvement of the pyroptosis pathway.

Another cell death pathway, necroptosis, which is a programmed form of necrosis or inflammatory cell death, has to date not been fully investigated in the brain upon infection with neurotropic viruses. A study by Liu et al. has shown that intra-cerebroventricular injection of TNF-α led to activation of RIP3-mediated necroptosis in hippocampal neurons, demonstrating that this pathway is functional in the brain (Liu et al., 2014). The observation that several components of the necroptosis pathway were more up-regulated in our microarray during the late stage of WNV and CHIKV infection compared to the early stage suggests a potential role for this pathway in the exacerbation of the outcome of neuroinvasive disease. However, as genes can have many different functions in multiple pathways and precise differences between different types of cell death are not well-defined, we cannot state with certainty that the pathways that we have identified in our study, such as pyroptosis and necroptosis, indeed play an important role in viral pathogenesis. As a result, future experiments measuring activation of both the pyroptosis and necroptosis pathway in both neuronal cell cultures and in the brains of adult mice upon WNV infection, as well as concomitant elimination experiments, should be carried out.

Although infection of a neuron often leads to cell death, it is possible that a phase of dysfunction precedes this stage of cell death. Using a curated set of genes, we did not find evidence for neuronal or mitochondrial dysfunction in either the WNV or the CHIKV infection model. However, we cannot rule out that our curated pathways did not contain all the relevant genes, nor that the analysis of bulk brain reduced the sensitivity of detecting dysfunction, consequently influencing the results. Furthermore, gene expression might not be the most suitable method for detecting cellular dysfunction, and further studies involving proteomics or IHC stainings may be more appropriate.

The transcript profiles obtained from mice during the late stage of WNV and CHIKV infection indicated a strong pro-inflammatory response, which is common during infection with both viruses. Many differentially expressed genes, but also many cytokines and immune pathways were found to be shared by CHIKV and WNV infection. Similarly, using meta-analysis, we observed a large overlap between our WNV and CHIKV transcriptome signatures and reovirus, JEV, and a virulent and avirulent strain of WNV, in particular at the early time points. At the late time point, CHIKV tended to have a slightly different profile when compared to other viruses, which suggests that CHIKV could have somewhat distinct mechanisms of pathogenesis and/or activated pathways as compared to the other viruses.

Several molecules that increase the inflammatory response in the brain, such as TNFRSF1A, CXCR4, CD47, and MAPK12, were up-regulated in the brains of both WNV and CHIKV infected mice. In addition, many transcription factors, such as NFκB, USF1, and FOX important for promoting inflammation, were found elevated in both our models. However, it is not clear whether the inflammatory response triggered during infection with WNV or CHIKV is an unsuccessful attempt to clear the infection with no share in the pathogenesis process or causally associated with disease. Even though it is very difficult to establish causality between neuroinflammation and pathology in primarily descriptive studies such as ours, there is circumstantial evidence for a role of neuroinflammation in disease.

For example, an excessive or dysregulated inflammatory response in the brain has been shown to be associated with both biological and clinical features of non-infectious neurologic disease (Gibney and Drexhage, 2013). Some cytokines such as IL-1β and TNF-ɑ may cause damage and death because of their neurotoxic activity through elevated glutamate production. TNF-ɑ has in fact been implicated in neuronal death by damaging mitochondria in neurons and increasing the production of ROS. Although TNF-ɑ was highly up-regulated in our study, no effect was seen in terms of mitochondrial dysfunction. Instead, it is possible that the effect on the mice in our study was restricted to mitochondria-induced apoptosis rather than dysfunction. Also chemokines were found to be highly up-regulated in our study during WNV and CHIKV infection, which are able to induce neuronal death directly through the activation of neuronal chemokine receptors or indirectly through the activation of microglial killing mechanisms.

Neurodegeneration has been linked to several mediators, such as IL-1β, IL-6, IL-8, IL-33, TNF-α, CCL2, CCL5, MMPs, reactive oxygen species (ROS), CD40, CD40L, CD88, and MAPKs (Ke et al., 2005; Ager et al., 2010; Glass et al., 2010; Kim and Choi, 2010; Kim and Joh, 2012; Amor et al., 2014; Skaper et al., 2014; Kempuraj et al., 2015). As we were interested in determining whether the inflammatory response during the late stage of WNV and CHIKV neuroinvasive disease is similar to that seen in neurodegenerative diseases, we specifically compared our data with such known mediators. In line with what has been found in several neurodegenerative diseases, many of these inflammatory mediators were found differentially regulated in our study, including CXCL10, a chemokine shown to induce apoptosis in fetal neurons (Sui et al., 2004). Taken together, these mediators may provide the link between inflammation and cell death.

Frasier et al. analyzed protein expression during the late and early phase of neuroinvasive disease of mice infected with WNV and CHIKV using 2D-DIGE and iTRAQ analysis (Fraisier et al., 2013). Comparing the late time point with the early for WNV, they were able to detect up-regulation of a few proteins, including Cbr1, Hspa2, Vps45, Hba-a1, Ranbp3 and Ahsg, as well as down-regulation of Crmp1 and Araf. These findings are not entirely supported by our gene expression results, however, as Crmp1 was the only detected protein that corresponded with our gene expression results. Nonetheless, this discordance is most likely caused by the difference in kinetics of mRNA and protein expression as they are often not highly correlated due to the influence of, for example, post-transcriptional/translational regulations.

In conclusion, this study provides evidence for the involvement of cell death pathways such as apoptosis and pyroptosis during WNV and CHIKV neuroinvasive disease, with some evidence of involvement of necroptosis during WNV severe disease. In addition, a strong inflammatory response was found in mice infected with WNV and CHIKV, as well as some overlap with inflammatory markers found in neurodegenerative diseases. Additional *in vitro* and *in vivo* studies should be conducted to further validate activation of cell death pathways during infection, including autophagy, as well as their contribution to the outcome of neuroinvasive disease. Identifying pathways or markers of viral neuroinvasive disease, which overlap with other non-infectious neurodegenerative diseases, may be useful for finding potential targets for treatment of neurologic diseases.

## Author contributions

BM, PK, and AO designed the experiments; SL, PK, BM, MO, EM, FZ, and JR performed the experiments; SL, Hv, MO, PK, BM, Wv, and AA analyzed the data. SL, Hv, BM, PK, AA, and AO wrote and revised the manuscript.

### Conflict of interest statement

The authors declare that the research was conducted in the absence of any commercial or financial relationships that could be construed as a potential conflict of interest. The reviewer HL and handling Editor declared their shared affiliation, and the handling Editor states that the process nevertheless met the standards of a fair and objective review.

## References

[B1] Abraham R. Mudaliar P. Padmanabhan A. Sreekumar E. (2013). Induction of cytopathogenicity in human glioblastoma cells by chikungunya virus. PLoS ONE 8:e75854. 10.1371/journal.pone.0075854 24086645 PMC3783433

[B2] Ager R. R. Fonseca M. I. Chu S. H. Sanderson S. D. Taylor S. M. Woodruff T. M.. (2010). Microglial C5aR (CD88) expression correlates with amyloid-beta deposition in murine models of Alzheimer's disease. J. Neurochem. 113, 389–401. 10.1111/j.1471-4159.2010.06595.x 20132482 PMC2921960

[B3] Ali Ou Alla S. Combe B. (2011). Arthritis after infection with Chikungunya virus. Best Pract. Res. Clin. Rheumatol. 25, 337–346. 10.1016/j.berh.2011.03.005 22100284

[B4] Amor S. Peferoen L. A. Vogel D. Y. Breur M. van der Valk P. Baker D.. (2014). Inflammation in neurodegenerative diseases–an update. Immunology 142, 151–166. 10.1111/imm.12233 24329535 PMC4008224

[B5] Barlan A. U. Griffin T. M. McGuire K. A. Wiethoff C. M. (2011). Adenovirus membrane penetration activates the NLRP3 inflammasome. J. Virol. 85, 146–155. 10.1128/JVI.01265-10 20980503 PMC3014182

[B6] Beatman E. Oyer R. Shives K. D. Hedman K. Brault A. C. Tyler K. L.. (2012). West Nile virus growth is independent of autophagy activation. Virology 433, 262–272. 10.1016/j.virol.2012.08.016 22939285 PMC3444629

[B7] Benjamini Y. Hochberg Y. (1995). Controlling the false discovery rate: a practical and powerful approach to multiple testing. J. R. Stat. Soc. B. 57, 289–300.

[B8] Bourgeois M. A. Denslow N. D. Seino K. S. Barber D. S. Long M. T. (2011). Gene expression analysis in the thalamus and cerebrum of horses experimentally infected with West Nile virus. PLoS ONE 6:e24371. 10.1371/journal.pone.0024371 21991302 PMC3186766

[B9] Burt F. J. Rolph M. S. Rulli N. E. Mahalingam S. Heise M. T. (2012). Chikungunya: a re-emerging virus. Lancet 379, 662–671. 10.1016/S0140-6736(11)60281-X 22100854

[B10] Campbell G. L. Marfin A. A. Lanciotti R. S. Gubler D. J. (2002). West Nile virus. *Lancet Infect*. Dis. 2, 519–529. 10.1016/S1473-3099(02)00368-7 12206968

[B11] Chandak N. H. Kashyap R. S. Kabra D. Karandikar P. Saha S. S. Morey S. H. (2009). Neurological complications of Chikungunya virus infection. *Neurol*. India 57, 177–180. 10.4103/0028-3886.51289 19439849

[B12] Cheeran M. C. Hu S. Sheng W. S. Rashid A. Peterson P. K. Lokensgard J. R. (2005). Differential responses of human brain cells to West Nile virus infection. *J*. Neurovirol. 11, 512–524. 10.1080/13550280500384982 16338745

[B13] Chen X. He W. T. Hu L. Li J. Fang Y. Wang X.. (2016). Pyroptosis is driven by non-selective gasdermin-D pore and its morphology is different from MLKL channel-mediated necroptosis. Cell Res. 26, 1007–1020. 10.1038/cr.2016.100 27573174 PMC5034106

[B14] Chiam C. W. Chan Y. F. Ong K. C. Wong K. T. Sam I. C. (2015). Neurovirulence comparison of chikungunya virus isolates of the Asian and East/Central/South African genotypes from Malaysia. J. Gen. Virol. 96, 3243–3254. 10.1099/jgv.0.000263 26276497

[B15] Chu J. J. Ng M. L. (2003). The mechanism of cell death during West Nile virus infection is dependent on initial infectious dose. J. Gen. Virol. 84, 3305–3314. 10.1099/vir.0.19447-0 14645911

[B16] Clarke P. Leser J. S. Bowen R. A. Tyler K. L. (2014a). Virus-induced transcriptional changes in the brain include the differential expression of genes associated with interferon, apoptosis, interleukin 17 receptor A, and glutamate signaling as well as flavivirus-specific upregulation of tRNA synthetases. MBio. 5:e00902–14. 10.1128/mBio.00902-14 24618253 PMC3952157

[B17] Clarke P. Leser J. S. Quick E. D. Dionne K. R. Beckham J. D. Tyler K. L. (2014b). Death receptor-mediated apoptotic signaling is activated in the brain following infection with West Nile virus in the absence of a peripheral immune response. J. Virol. 88, 1080–1089. 10.1128/JVI.02944-13 24198425 PMC3911655

[B18] Couderc T. Chretien F. Schilte C. Disson O. Brigitte M. Guivel-Benhassine F.. (2008). A mouse model for Chikungunya: young age and inefficient type-I interferon signaling are risk factors for severe disease. PLoS Pathog. 4:e29. 10.1371/journal.ppat.0040029 18282093 PMC2242832

[B19] Dantuma N. P. Bott L. C. (2014). The ubiquitin-proteasome system in neurodegenerative diseases: precipitating factor, yet part of the solution. Front. Mol. Neurosci. 7:70. 10.3389/fnmol.2014.00070 25132814 PMC4117186

[B20] Das T. Hoarau J. J. Jaffar Bandjee M. C. Maquart M. Gasque P. (2015). Multifaceted innate immune responses engaged by astrocytes, microglia and resident dendritic cells against Chikungunya neuroinfection. J. Gen. Virol. 96, 294–310. 10.1099/vir.0.071175-0 25351727

[B21] de Castro I. P. Martins L. M. Tufi R. (2010). Mitochondrial quality control and neurological disease: an emerging connection. Expert Rev. Mol. Med. 12:e12. 10.1017/S1462399410001456 20398440 PMC2871738

[B22] Dhanwani R. Khan M. Bhaskar A. S. Singh R. Patro I. K. Rao P. V.. (2012). Characterization of Chikungunya virus infection in human neuroblastoma SH-SY5Y cells: role of apoptosis in neuronal cell death. Virus Res. 163, 563–572. 10.1016/j.virusres.2011.12.009 22210004

[B23] Diallo M. Thonnon J. Traore-Lamizana M. Fontenille D. (1999). Vectors of Chikungunya virus in Senegal: current data and transmission cycles. Am. J. Trop. Med. Hyg. 60, 281–286. 10072152 10.4269/ajtmh.1999.60.281

[B24] Diniz J. A. Da Rosa A. P. Guzman H. Xu F. Xiao S. Y. Popov V. L.. (2006). West Nile virus infection of primary mouse neuronal and neuroglial cells: the role of astrocytes in chronic infection. Am. J. Trop. Med. Hyg. 75, 691–696. 17038696

[B25] Dupuis-Maguiraga L. Noret M. Brun S. Le Grand R. Gras G. Roques P. (2012). Chikungunya disease: infection-associated markers from the acute to the chronic phase of arbovirus-induced arthralgia. PLoS Negl. Trop. Dis. 6:e1446. 10.1371/journal.pntd.0001446 22479654 PMC3313943

[B26] Fabregat A. Sidiropoulos K. Garapati P. Gillespie M. Hausmann K. Haw R.. (2016). The Reactome pathway Knowledgebase. Nucleic Acids Res. 44, D481–D487. 10.1093/nar/gkv1351 26656494 PMC4702931

[B27] Fraisier C. Camoin L. Lim S. M. Bakli M. Belghazi M. Fourquet P.. (2013). Altered protein networks and cellular pathways in severe west Nile disease in mice. PLoS ONE 8:e68318. 10.1371/journal.pone.0068318 23874584 PMC3707916

[B28] Gautier L. Cope L. Bolstad B. M. Irizarry R. A. (2004). affy–analysis of Affymetrix genechip data at the probe level. Bioinformatics 20, 307–315. 10.1093/bioinformatics/btg405 14960456

[B29] Gerardin P. Couderc T. Bintner M. Tournebize P. Renouil M. Lemant J. (2016). Chikungunya virus-associated encephalitis: a cohort study on La Reunion Island, 2005-2009. Neurology 86, 94–102. 10.1212/WNL.0000000000002234 26609145

[B30] Ghavami S. Shojaei S. Yeganeh B. Ande S. R. Jangamreddy J. R. Mehrpour M.. (2014). Autophagy and apoptosis dysfunction in neurodegenerative disorders. Prog. Neurobiol. 112, 24–49. 10.1016/j.pneurobio.2013.10.004 24211851

[B31] Gibney S. M. Drexhage H. A. (2013). Evidence for a dysregulated immune system in the etiology of psychiatric disorders. J. Neuroimmune Pharmacol. 8, 900–920. 10.1007/s11481-013-9462-8 23645137

[B32] Glass C. K. Saijo K. Winner B. Marchetto M. C. Gage F. H. (2010). Mechanisms underlying inflammation in neurodegeneration. Cell. 140, 918–934. 10.1016/j.cell.2010.02.016 20303880 PMC2873093

[B33] Gosztonyi G. (2001). The mechanisms of neuronal damage in virus infections of the nervous system. Curr. Top. Microbiol. Immunol. 253, 1–273. 10.1007/978-3-662-10356-2 11417130

[B34] Guo H. Callaway J. B. Ting J. P. (2015). Inflammasomes: mechanism of action, role in disease, and therapeutics. Nat. Med. 21, 677–687. 10.1038/nm.3893 26121197 PMC4519035

[B35] Honig L. S. Rosenberg R. N. (2000). Apoptosis and neurologic disease. Am. J Med. 108, 317–330. 10.1016/S0002-9343(00)00291-6 11014725

[B36] Huang W. X. Huang P. Hillert J. (2004). Increased expression of caspase-1 and interleukin-18 in peripheral blood mononuclear cells in patients with multiple sclerosis. Mult. Scler. 10, 482–487. 10.1191/1352458504ms1071oa 15471361

[B37] Hussmann K. L. Samuel M. A. Kim K. S. Diamond M. S. Fredericksen B. L. (2013). Differential replication of pathogenic and nonpathogenic strains of West Nile virus within astrocytes. J. Virol. 87, 2814–2822. 10.1128/JVI.02577-12 23269784 PMC3571364

[B38] Joubert P. E. Werneke S. W. de la Calle C. Guivel-Benhassine F. Giodini A. Peduto L.. (2012). Chikungunya virus-induced autophagy delays caspase-dependent cell death. J. Exp. Med. 209, 1029–1047. 10.1084/jem.20110996 22508836 PMC3348111

[B39] Judith D. Mostowy S. Bourai M. Gangneux N. Lelek M. Lucas-Hourani M.. (2013). Species-specific impact of the autophagy machinery on Chikungunya virus infection. EMBO Rep. 14, 534–544. 10.1038/embor.2013.51 23619093 PMC3674439

[B40] Ke Z. J. Calingasan N. Y. DeGiorgio L. A. Volpe B. T. Gibson G. E. (2005). CD40-CD40L interactions promote neuronal death in a model of neurodegeneration due to mild impairment of oxidative metabolism. *Neurochem*. Int. 47, 204–215. 10.1016/j.neuint.2005.03.002 15885854

[B41] Kempuraj D. Thangavel R. Yang E. Pattani S. Zaheer S. Santillan D. A.. (2015). Dopaminergic Toxin 1-Methyl-4-phenylpyridinium, proteins α-synuclein and glia maturation factor activate mast cells and release inflammatory mediators. PLoS ONE 10:e0135776. 10.1371/journal.pone.0135776 26275153 PMC4537263

[B42] Kim E. K. Choi E. J. (2010). Pathological roles of MAPK signaling pathways in human diseases. Biochim. Biophys. Acta 1802, 396–405. 10.1016/j.bbadis.2009.12.009 20079433

[B43] Kim G. H. Kim J. E. Rhie S. J. Yoon S. (2015). The role of oxidative stress in neurodegenerative diseases. Exp. Neurobiol. 24, 325–340. 10.5607/en.2015.24.4.325 26713080 PMC4688332

[B44] Kim Y. S. Joh T. H. (2012). Matrix metalloproteinases, new insights into the understanding of neurodegenerative disorders. *Biomol*. Ther. 20, 133–143. 10.4062/biomolther.2012.20.2.133 PMC3792209 24116286

[B45] Kleinschmidt M. C. Michaelis M. Ogbomo H. Doerr H. W. Cinatl J. Jr. (2007). Inhibition of apoptosis prevents West Nile virus induced cell death. BMC Microbiol. 7:49. 10.1186/1471-2180-7-49 17535425 PMC1891299

[B46] Kobayashi S. Orba Y. Yamaguchi H. Takahashi K. Sasaki M. Hasebe R.. (2014). Autophagy inhibits viral genome replication and gene expression stages in West Nile virus infection. Virus Res. 191, 83–91. 10.1016/j.virusres.2014.07.016 25091564

[B47] Koyuncu O. O. Hogue I. B. Enquist L. W. (2013). Virus infections in the nervous system. Cell Host Microbe 13, 379–393. 10.1016/j.chom.2013.03.010 23601101 PMC3647473

[B48] Krejbich-Trotot P. Denizot M. Hoarau J. J. Jaffar-Bandjee M. C. Das T. Gasque P. (2011a). Chikungunya virus mobilizes the apoptotic machinery to invade host cell defenses. FASEB J. 25, 314–325. 10.1096/fj.10-164178 20881210

[B49] Krejbich-Trotot P. Gay B. Li-Pat-Yuen G. Hoarau J. J. Jaffar-Bandjee M. C. Briant L. (2011b). Chikungunya triggers an autophagic process which promotes viral replication. *Virol*. J. 8:432 10.1186/1743-422X-8-432 PMC3179960 21902836

[B50] Kumar M. Belcaid M. Nerurkar V. R. (2016). Identification of host genes leading to West Nile virus encephalitis in mice brain using RNA-seq analysis. Sci. Rep. 6:26350. 10.1038/srep26350 27211830 PMC4876452

[B51] Lewthwaite P. Vasanthapuram R. Osborne J. C. Begum A. Plank J. L. Shankar M. V.. (2009). Chikungunya virus and central nervous system infections in children, India. Emerg. Infect. Dis. 15, 329–331. 10.3201/eid1502.080902 19193287 PMC2662654

[B52] Lim S. M. Koraka P. van Boheemen S. Roose J. M. Jaarsma D. van de Vijver D. A.. (2013). Characterization of the mouse neuroinvasiveness of selected European strains of West Nile virus. PLoS ONE 8:e74575. 10.1371/journal.pone.0074575 24058590 PMC3776840

[B53] Liu S. Wang X. Li Y. Xu L. Yu X. Ge L.. (2014). Necroptosis mediates TNF-induced toxicity of hippocampal neurons. Biomed. Res. Int. 2014:290182. 10.1155/2014/290182 25093162 PMC4100394

[B54] Losy J. Niezgoda A. (2001). IL-18 in patients with multiple sclerosis. Acta Neurol. Scand. 104, 171–173. 10.1034/j.1600-0404.2001.00356.x 11551238

[B55] Matthews V. Robertson T. Kendrick T. Abdo M. Papadimitriou J. McMinn P. (2000). Morphological features of Murray Valley encephalitis virus infection in the central nervous system of Swiss mice. Int. J. Exp. Pathol. 81, 31–40. 10.1046/j.1365-2613.2000.00135.x 10718862 PMC2517828

[B56] Mattson M. P. (2000). Apoptosis in neurodegenerative disorders. Nat. Rev. Mol. Cell Biol. 1, 120–129. 10.1038/35040009 11253364

[B57] Millecamps S. Julien J. P. (2013). Axonal transport deficits and neurodegenerative diseases. Nat. Rev. Neurosci. 14, 161–176. 10.1038/nrn3380 23361386

[B58] Munoz-Erazo L. Natoli R. Provis J. M. Madigan M. C. King N. J. (2012). Microarray analysis of gene expression in West Nile virus-infected human retinal pigment epithelium. Mol. Vis. 18, 730–743. 22509103 PMC3324360

[B59] Myint K. S. Kipar A. Jarman R. G. Gibbons R. V. Perng G. C. Flanagan B.. (2014). Neuropathogenesis of Japanese encephalitis in a primate model. PLoS. Negl. Trop. Dis. 8:e2980. 10.1371/journal.pntd.0002980 25102067 PMC4125110

[B60] Nayak T. K. Mamidi P. Kumar A. Singh L. P. Sahoo S. S. Chattopadhyay S.. (2017). Regulation of viral replication, apoptosis and pro-inflammatory responses by 17-AAG during Chikungunya virus infection in macrophages. Viruses 9:3. 10.3390/v9010003 28067803 PMC5294972

[B61] Parquet M. C. Kumatori A. Hasebe F. Mathenge E. G. Morita K. (2002). St. Louis encephalitis virus induced pathology in cultured cells. Arch. Virol. 1105–1119. 10.1007/s00705-002-0806-6 12111422

[B62] Parquet M. C. Kumatori A. Hasebe F. Morita K. Igarashi A. (2001). West Nile virus-induced bax-dependent apoptosis. FEBS Lett. 500, 17–24. 10.1016/S0014-5793(01)02573-X 11434919

[B63] Peter R. Krishnan L. Anandraj V. Kuruvila S. (2015). Chikungunya in a newborn. J. Clin. Neonatol. 4, 145–146. 10.4103/2249-4847.154134

[B64] Petersen L. R. Marfin A. A. (2002). West Nile virus: a primer for the clinician. Ann. Intern. Med. 137, 173–179. 10.7326/0003-4819-137-3-200208060-00009 12160365

[B65] Phipson B. Lee S. Majewski I. J. Alexander W. S. Smyth G. K. (2016). Robust hyperparameter estimation protects against hypervariable genes and improves power to detect differential expression. Ann. Appl. Stat. 10, 946–963. 10.1214/16-AOAS920 28367255 PMC5373812

[B66] Poo Y. S. Rudd P. A. Gardner J. Wilson J. A. Larcher T. Colle M. A.. (2014). Multiple immune factors are involved in controlling acute and chronic chikungunya virus infection. PLoS Negl. Trop. Dis. 8:e3354. 10.1371/journal.pntd.0003354 25474568 PMC4256279

[B67] Ritchie M. E. Phipson B. Wu D. Hu Y. Law C. W. Shi W.. (2015). limma powers differential expression analyses for RNA-sequencing and microarray studies. Nucleic Acids Res. 43:e47. 10.1093/nar/gkv007 25605792 PMC4402510

[B68] Rossi S. L. Ross T. M. Evans J. D. (2010). West Nile virus. Clin. Lab. Med. 30, 47–65. 10.1016/j.cll.2009.10.006 20513541 PMC2905782

[B69] Saco T. Parthasarathy P. T. Cho Y. Lockey R. F. Kolliputi N. (2014). Inflammasome: a new trigger of Alzheimer's disease. Front. Aging Neurosci. 6:80. 10.3389/fnagi.2014.00080 24834051 PMC4018519

[B70] Samuel M. A. Morrey J. D. Diamond M. S. (2007). Caspase 3-dependent cell death of neurons contributes to the pathogenesis of West Nile virus encephalitis. J. Virol. 81, 2614–2623. 10.1128/JVI.02311-06 17192305 PMC1866006

[B71] Scheper W. Hoozemans J. J. (2015). The unfolded protein response in neurodegenerative diseases: a neuropathological perspective. Acta Neuropathol. 130, 315–331. 10.1007/s00401-015-1462-8 26210990 PMC4541706

[B72] Schon E. A. Manfredi G. (2003). Neuronal degeneration and mitochondrial dysfunction. J. Clin. Invest. 111, 303–312. 10.1172/JCI17741 12569152 PMC151870

[B73] Sejvar J. J. (2014). Clinical manifestations and outcomes of West Nile virus infection. Viruses. 6, 606–623. 10.3390/v6020606 24509812 PMC3939474

[B74] Shrestha B. Gottlieb D. Diamond M. S. (2003). Infection and injury of neurons by West Nile encephalitis virus. J. Virol. 77, 13203–13213. 10.1128/JVI.77.24.13203-13213.2003 14645577 PMC296085

[B75] Skaper S. D. Facci L. Giusti P. (2014). Mast cells, glia and neuroinflammation: partners in crime? Immunology 141, 314–327. 10.1111/imm.12170 24032675 PMC3930370

[B76] Sui Y. Potula R. Dhillon N. Pinson D. Li S. Nath A.. (2004). Neuronal apoptosis is mediated by CXCL10 overexpression in simian human immunodeficiency virus encephalitis. Am. J. Pathol. 164, 1557–1566. 10.1016/S0002-9440(10)63714-5 15111302 PMC1615658

[B77] Sutinen E. M. Pirttila T. Anderson G. Salminen A. Ojala J. O. (2012). Pro-inflammatory interleukin-18 increases Alzheimer's disease-associated amyloid-beta production in human neuron-like cells. J. Neuroinflammation. 9:199. 10.1186/1742-2094-9-199 22898493 PMC3458954

[B78] The Gene Ontology, C. (2017). Expansion of the Gene Ontology knowledgebase and resources. Nucleic Acids Res. 45, D331–D338. 10.1093/nar/gkw1108 27899567 PMC5210579

[B79] Tournebize P. Charlin C. Lagrange M. (2009). Neurological manifestations in Chikungunya: about 23 cases collected in Reunion Island. Rev. Neurol. 165, 48–51. 10.1016/j.neurol.2008.06.009 18835614

[B80] Troulinaki K. Tavernarakis N. (2012). Necrotic cell death and neurodegeneration: The involvement of endocytosis and intracellular trafficking. Worm 1, 176–181. 10.4161/worm.20457 24058844 PMC3670410

[B81] Tselis A. Booss J. (2014). Handbook of Clinical Neurology, 1st Edn. Amsterdam: Elsevier.

[B82] van den Doel P. Volz A. Roose J. M. Sewbalaksing V. D. Pijlman G. P. van Middelkoop I.. (2014). Recombinant modified vaccinia virus Ankara expressing glycoprotein E2 of Chikungunya virus protects AG129 mice against lethal challenge. PLoS Negl. Trop. Dis. 8:e3101. 10.1371/journal.pntd.0003101 25188230 PMC4154657

[B83] Vandergaast R. Fredericksen B. L. (2012). West Nile virus (WNV) replication is independent of autophagy in mammalian cells. PLoS ONE 7:e45800. 10.1371/journal.pone.0045800 23029249 PMC3448696

[B84] Venter M. Myers T. G. Wilson M. A. Kindt T. J. Paweska J. T. Burt F. J.. (2005). Gene expression in mice infected with West Nile virus strains of different neurovirulence. Virology 342, 119–140. 10.1016/j.virol.2005.07.013 16125213

[B85] Wang W. Bu B. Xie M. Zhang M. Yu Z. Tao D. (2009). Neural cell cycle dysregulation and central nervous system diseases. *Prog*. Neurobiol. 89, 1–17. 10.1016/j.pneurobio.2009.01.007 19619927

[B86] Wu M. F. Chen S. T. Yang A. H. Lin W. W. Lin Y. L. Chen N. J.. (2013). CLEC5A is critical for dengue virus-induced inflammasome activation in human macrophages. Blood 121, 95–106. 10.1182/blood-2012-05-430090 23152543

[B87] Yang J. S. Ramanathan M. P. Muthumani K. Choo A. Y. Jin S. H. Yu Q. C.. (2002). Induction of inflammation by West Nile virus capsid through the caspase-9 apoptotic pathway. Emerg. Infect. Dis. 8, 1379–1384. 10.3201/eid0812.020224 12498651 PMC2738518

[B88] Yang M. R. Lee S. R. Oh W. Lee E. W. Yeh J. Y. Nah J. J.. (2008). West Nile virus capsid protein induces p53-mediated apoptosis via the sequestration of HDM2 to the nucleolus. Cell Microbiol. 10, 165–176. 10.1111/j.1462-5822.2007.01027.x 17697133 PMC7162166

