# Supplementary material for: Transcriptomic Analyses Reveal Differential Gene Expression of Immune and Cell Death Pathways in the Brains of Mice Infected with West Nile Virus and Chikungunya Virus
Source: Front Microbiol. 2017 Aug 17;8:1556. doi: 10.3389/fmicb.2017.01556 (PMC5562671; doi:10.3389/fmicb.2017.01556)
Supplement: Supplementary file 2 [file Table2.DOCX]

| **Cytokines** | | **WNV-L vs WNV-E** | **CHIKV-L vs CHIKV-E** |
| --- | --- | --- | --- |
| **Symbol** | **Entrez Gene Name** | **Log_2_ ratio fold change** | **Log_2_ ratio fold change** |
| IL1B | Interleukin 1beta | 0.75 | 1.31 |
| IL6 | Interleukin 6 | 3.62 | 2.51 |
| TNF | Tumor necros factor | 1.17 | 0.64 |
| IFNG | Interferon gamma | 2.56 | 0.43 |
| IL18 | Interleukin 18 | 0 | 0 |
| IL17A | Interleukin 17A | 0 | 0 |
| IL23 | Interleukin 23 | 0 | 0 |
| IL12A | Interleukin 12A | 0 | 0 |
| IL12B | Interleukin 12B | 2.24 | 1.16 |
| IL10 | Interleukin 10 | 0.55 | 0.21 |
| C3 | Complement component 3 | 3.52 | 1.20 |
| C5 | Complement component 5 | 0 | 0.22 |

**Table S2.** Differential gene expression of cytokines at the late stage of WNV and CHIKV infection compared to early.
