# Supplementary material for: Transcriptomic Analyses Reveal Differential Gene Expression of Immune and Cell Death Pathways in the Brains of Mice Infected with West Nile Virus and Chikungunya Virus
Source: Front Microbiol. 2017 Aug 17;8:1556. doi: 10.3389/fmicb.2017.01556 (PMC5562671; doi:10.3389/fmicb.2017.01556)
Supplement: Supplementary file 3 [file Table3.DOCX]

**Table S3.** Differential gene expression of receptors and regulatory proteins at the late stage of WNV and CHIKV infection compared to early.

| **Receptors and Regulatory Proteins** | | **WNV-L vs WNV-E** | **CHIKV-L vs CHIKV-E** |
| --- | --- | --- | --- |
| **Symbol** | **Entrez Gene Name** | **Log_2_ ratio fold change** | **Log_2_ ratio fold change** |
| TNFRSF1A | TNF receptor superfamily member 1a | 0.79 | 1.15 |
| TNFRSF1B | TNF receptor superfamily member 1b | 0.70 | 1.03 |
| CXCR4 | C-X-C chemokine receptor type 4 | 0.42 | 0.65 |
| CD22 | Cluster of differentiation-22 | -0.25 | 0 |
| CD47 | Cluster of differentiation-47 | 0.45 | 0.49 |
| CD200 | Cluster of differentiation-200 | 0 | 0 |
| ICAM5 | Intercellular adhesion molecule 5 | 0 | 0.68 |
| NCAM1 | Neural cell adhesion molecule 1 | -0.46 | -0.44 |
| NCAM2 | Neural cell adhesion molecule 2 | 0 | -0.31 |
| HMGB1 | High mobility group box protein 1 | 0 | 0 |
| TXN | Thioredoxin | 0 | 0.47 |
| TXN2 | Thioredoxin 2 | -0.33 | 0 |
| SEMA3A | Semaphorin 3A | 0 | 0 |
| SEMA3B | Semaphorin 3B | -0.19 | 0 |
| SEMA3C | Semaphorin 3C | -0.44 | -0.27 |
| SEMA3D | Semaphorin 3D | 0 | 0 |
| SEMA3E | Semaphorin 3E | 0 | -0.93 |
| SEMA3F | Semaphorin 3F | 0 | 0 |
| SEMA3G | Semaphorin 3G | 0 | 0 |
| SEMA4A | Semaphorin 4A | -0.23 | 0 |
| SEMA4B | Semaphorin 4B | 0 | 0 |
| SEMA4C | Semaphorin 4C | 0 | 0.36 |
| SEMA4D | Semaphorin 4D | 0 | 0.46 |
| SEMA4F | Semaphorin 4F | 0 | -0.37 |
| SEMA4G | Semaphorin 4G | -0.67 | 0 |
| SEMA5A | Semaphorin 5A | 0 | -0.66 |
| SEMA5B | Semaphorin 5B | 0 | 0 |
| SEMA6A | Semaphorin 6A | -0.60 | -0.68 |
| SEMA6B | Semaphorin 6B | 0 | 0 |
| SEMA6C | Semaphorin 6C | -0.41 | 0 |
| SEMA6D | Semaphorin 6D | -0.24 | -1.04 |
| SEMA7A | Semaphorin 7A | 0 | 0 |
| MAPK1 | Mitogen activated protein kinase 1 | 0.62 | 0 |
| MAPK3 | Mitogen activ44ated protein kinase 3 | -0.45 | 0 |
| MAPK7 | Mitogen activated protein kinase 7 | 0 | -0.28 |
| MAPK8 | Mitogen activated protein kinase 8 | 0.36 | 0 |
| MAPK9 | Mitogen activated protein kinase 9 | 0 | -0.44 |
| MAPK10 | Mitogen activated protein kinase 10 | 0 | 0 |
| MAPK11 | Mitogen activated protein kinase 11 | 0 | -0.37 |
| MAPK12 | Mitogen activated protein kinase 12 | 0.32 | 0.33 |
| MAPK13 | Mitogen activated protein kinase 13 | -0.20 | 0 |
| MAPK14 | Mitogen activated protein kinase 14 | -0.23 | -0.25 |
| MAPK8IP1 | Mitogen activated protein kinase 8 interacting protein 1 | -0.60 | 0 |
| MAPKAP2 | Mitogen activated protein kinase-activated protein kinase 2 | 0 | 0.92 |
| MAPKAP3 | Mitogen activated protein kinase-activated protein kinase 3 | 1.40 | 0.63 |
| NOS1 | Nitric oxide synthase 1 | -0.50 | 0 |
| NOS2 | Nitric oxide synthase 2 | 0 | 0.28 |
| NOS3 | Nitric oxide synthase 3 | -0.33 | 0 |
| NFkB1 | Nuclear factor kappa B subunit 1 | 0.77 | 0 |
| NFkB2 | Nuclear factor kappa B subunit 2 | 0.96 | 1.02 |
| USF1 | Upstream stimulatory factor 1 | 0 | 0.58 |
| SP1 | Specificity protein 1 | 0.31 | -0.29 |
| FOXA2 | Forkhead box A2 | 0 | 0 |
| FOXJ1 | Forkhead box J1 | 0 | 0.36 |
| FOXL2 | Forkhead box L2 | -0.38 | 0.20 |
| FOXN1 | Forkhead box N1 | 0 | 0 |
| FOXO1 | Forkhead box O1 | -0.24 | 0.29 |
| FOXO3 | Forkhead box O3 | 0.32 | 0.50 |
| FOXO4 | Forkhead box O4 | -0.28 | 0 |
| FOXP3 | Forkhead box P3 | 0 | 0 |
| XBP1 | X-box binding protein 1 | 0.51 | 0 |
