# Supplementary material for: Transcriptomic Analyses Reveal Differential Gene Expression of Immune and Cell Death Pathways in the Brains of Mice Infected with West Nile Virus and Chikungunya Virus
Source: Front Microbiol. 2017 Aug 17;8:1556. doi: 10.3389/fmicb.2017.01556 (PMC5562671; doi:10.3389/fmicb.2017.01556)
Supplement: Supplementary file 4 [file Table4.DOCX]

| **Chemokines** | | **WNV-L vs WNV-E** | **CHIKV-L vs CHIKV-E** |
| --- | --- | --- | --- |
| **Symbol** | **Entrez Gene Name** | **Log_2_ ratio fold change** | **Log_2_ ratio fold change** |
| CX3CL1 | C-X3-C motif chemokine ligand 1 | -0.41 | 0.46 |
| CXCL10 | C-X-C motif chemokine ligand 10 | 2.10 | 5.09 |
| CXCR2 | C-X-C motif chemokine receptor type 2 | 0 | 0 |
| CXCL12 | C-X-C motif chemokine ligand 12 | -1.01 | -1.10 |
| CCL2 | C-C motif chemokine ligand 2 | 3.90 | 3.61 |
| CCL3 | C-C motif chemokine ligand 3 | 3.07 | 3.19 |
| CXCL2 | C-X-C motif chemokine ligand 2 | 3.24 | 1.71 |
| CCL5 | C-C motif chemokine ligand 5 | 4.48 | 4.00 |
| MIF | Macrophage migration inhibitory factor | -0.37 | 0 |
| CCL8 | C-C motif chemokine ligand 8 | 0.28 | 0.49 |
| CCL20 | C-C motif chemokine ligand 20 | 0 | 0 |
| CXCL1 | C-X-C motif chemokine ligand 1 | 3.33 | 2.42 |
| CCL4 | C-C motif chemokine ligand 4 | 2.53 | 2.41 |
| CCL9 | C-C motif chemokine ligand 9 | 0.97 | 0.98 |
| XCL1 | X-C motif chemokine ligand 1 | 0 | 0 |
| CCL1 | C-C motif chemokine ligand 1 | 0 | 0 |
| CCL11 | C-C motif chemokine ligand 11 | 0.49 | 0.49 |
| CCL24 | C-C motif chemokine ligand 24 | 0 | 0 |
| CCL25 | C-C motif chemokine ligand 25 | 0.35 | 0.35 |
| CXCL13 | C-X-C motif chemokine ligand 13 | 1.82 | 2.09 |

**Table S4.** Differential gene expression of chemokines at the late stage of WNV and CHIKV infection compared to early.
