# Supplementary material for: Transcriptomic Analyses Reveal Differential Gene Expression of Immune and Cell Death Pathways in the Brains of Mice Infected with West Nile Virus and Chikungunya Virus
Source: Front Microbiol. 2017 Aug 17;8:1556. doi: 10.3389/fmicb.2017.01556 (PMC5562671; doi:10.3389/fmicb.2017.01556)
Supplement: Supplementary file 5 [file Table5.DOCX]

**Table S5.** Differential expression of genes involved in mitochondrial dysfunction at the late stage of WNV and CHIKV infection compared to early.

| **Mitochondrial dysfunction** | | **WNV-L vs WNV-E** | **CHIKV-L vs CHIKV-E** |
| --- | --- | --- | --- |
| **Symbol** | **Entrez Gene Name** | **Log_2_ ratio fold change** | **Log_2_ ratio fold change** |
| ITGAV | Integrin subunit alpha V | 0 | 0 |
| ITGB5 | Integrin subunit beta 5 | -0.5 | -0.58 |
| CLU | Clusterin | 0.70 | 0 |
| FBLN1 | Fibulin 1 | -0.20 | 0.42 |
| FBLN2 | Fibulin 2 | -0.42 | -0.49 |
| FBLN5 | Fibulin 5 | -0.43 | -0.35 |
| FBLN7 | Fibulin 7 | -0.61 | 0 |
| VTN | Vitronectin | -0.30 | 0 |
| COL1A1 | Collagen type I alpha 1 chain | -1.01 | 0 |
| COL5A1 | Collagen type V alpha 1 chain | 0 | -0.35 |
| MMP2 | Matrix metalloproteinase-2 | -0.25 | 0 |
| PLAU | Plasminogen activator urokinase | 0 | 0.37 |
| PLAUR | Plasminogen activator urokinase receptor | 0.70 | 0.88 |
| LOX | Lysyl oxidase | 0 | 0 |
| TIMP3 | TIMP metallopeptidase inhibitor 3 | 0.55 | 0 |
| APOE | Apolipoprotein E | -0.31 | -0.31 |
| VLDLR | Very low density lipoprotein receptor | 0.27 | -0.77 |
| CD68 | Cluster of differentiation-68 | 0.72 | 0.63 |
| SCARB1 | Scavenger receptor class B member 1 | -0.45 | 0 |
| SCARB2 | Scavenger receptor class B member 2 | -0.23 | 0.30 |
| LDLR | Low density lipoprotein receptor | -0.55 | -0.35 |
| TGFB1 | Transforming growth factor beta 1 | -0.22 | 2.76 |
| FGF5 | Fibroblast growth factor 5 | 0 | 0 |
| CD97 | Cluster of differentiation-97 | -0.18 | 0 |
| VEGFa | Vascular endothelial growth factor A | 0.49 | -0.52 |
| EPHX1 | Epoxide hydrolase 1 | 0.60 | 0 |
| MGST1 | Microsomal glutathione S-transferase 1 | 1.46 | 0.84 |
| SOD2 | Superoxide dismutase 2 | 1.22 | -0.50 |
| TOR1AIP1 | Torsin 1A interacting protein 1 | 0.59 | 0 |
| TOR1AIP2 | Torsin 1A interacting protein 2 | 0.92 | 0.86 |
| TOR1A | Torsin family 1 member A | 0.27 | 0 |
| TOR1B | Torsin family 1 member B | -0.25 | 0 |
| KTN1 | Kinectin 1 | 0 | 0 |
| CCT7 | Chaperonin containing TCP1 subunit 7 | 0 | 0 |
| LAMP1 | Lysosomal-associated membrane protein 1 | 0 | 0 |
| SLC20A2 | Solute carrier family 20 member 2 | 0 | 0 |
| MGAT2 | Mannosyl (alpha-1,6-)-glycoprotein beta-1,2-N-acetylglucosaminyltransferase | 0 | 0 |
| HK1 | Hexokinase 1 | -0.34 | 0 |
| HK2 | Hexokinase 2 | 1.11 | 0.32 |
| DNAJC1 | DnaJ heat shock protein family (Hsp40) member C1 | 0.84 | 0 |
| LDHB | L-lactate dehydrogenase B | 0 | 0 |
| HIF1A | Hypoxia inducible factor 1-alpha | 0.51 | 0 |
| EDN1 | Endothelin 1 | 0.19 | 0.43 |
| EDN2 | Endothelin 2 | 0 | 0 |
| EDN3 | Endothelin 3 | -0.46 | 0 |
| CAV1 | Caveolin 1 | -1.01 | -0.72 |
| ENG | Endoglin | 0 | 0.39 |
| TNFA | Tumor necrosis factor alpha | 1.17 | 0.73 |
| IL6 | Interleukin 6 | 3.63 | 3.10 |
| SLC25A4 | Solute carrier family 25 member 4 | 0 | 0 |
| GAPDH | Glyceraldehyde 3-phosphate dehydrogenase | 0 | 0.26 |
| COX6A1 | Cytochrome c oxidase subunit 6A1 | 0 | 0.32 |
| COX6A2 | Cytochrome c oxidase subunit 6A2 | 0.48 | 0 |
| CDKN1A | Cyclin dependent kinase inhibitor 1A | 0.97 | 1.11 |
| CDKN1C | Cyclin dependent kinase inhibitor 1C | -0.57 | 0 |
| CFLAR | Casp8 and FADD like apoptosis regulator | 1.40 | 1.43 |
| PEX6 | Peroxisomal biogenesis factor 6 | -0.68 | 0 |
| MAOA | Monoamine oxidase A | 0 | 0 |
| MTHFD1 | Methylenetetrahydrofolate dehydrogenase, cyclohydrolase and formyltetrahydrofolate synthetase 1 | 0 | -0.43 |
| MTHFD2 | Methylenetetrahydrofolate dehydrogenase, cyclohydrolase and formyltetrahydrofolate synthetase 2 | 1.80 | 0.45 |
| RAF1 | Raf-1 proto-oncogene | 0.29 | 0 |
| HAX1 | HCLS1 associated protein X-1 | 0.86 | 0 |
| TFAM | Transcription factor A mitochondrial | 0.78 | 0 |
| TFB1M | Transcription factor B1 mitochondrial | 0 | 0 |
| CytC | Cytochrome c | -0.30 | 0 |
| NFkB1 | Nuclear factor kappa B subunit 1 | 0.77 | 0 |
| NFKB2 | Nuclear factor kappa B subunit 2 | 0.96 | 1.02 |
| BNIP3 | BCL2 interacting protein 3 | 0 | 0 |
| PDK1 | Pyruvate dehydrogenase kinase 1 | 0.93 | 0.39 |
| MTTP | Microsomal triglyceride transfer protein | 0 | 0 |
| STAT1 | Signal transducer and activator of transcription 1 | 0 | 1.80 |
| TNFRSF14 | TNF receptor superfamily member 14 | 0 | 0 |
| APOL6 | Apolipoprotein L6 | 0 | 0.34 |
| PARP14 | Poly(ADP-ribose) polymerase family member 14 | 1.43 | 1.95 |
| PARP9 | Poly(ADP-ribose) polymerase family member 9 | 0.61 | 1.92 |
| A2M | Alpha-2-macroglobulin | 1.55 | 1.06 |
| FBXO6 | F-Box protein 6 | 0.51 | 0.35 |
| LHPP | Phospholysine phosphohistidine inorganic pyrophosphate phosphatase | -0.32 | 0 |
| NDUFS6 | NADH:ubiquinone oxidoreductase subunit S6 | 0 | 0 |
| NDUFV1 | NADH:ubiquinone oxidoreductase core subunit V1 | 0 | 0 |
| COX5A | Cytochrome C oxidase subunit 5A | -0.24 | 0 |
| COX6C | Cytochrome C oxidase subunit 6C | 0 | 0 |
| NDUFB8 | NADH:ubiquinone oxidoreductase subunit B8 | -0.26 | 0 |
| UQCRH | Ubiquinol-cytochrome c reductase hinge protein | -0.55 | 0 |
| NDUFAB1 | NADH:ubiquinone oxidoreductase subunit AB1 | -0.39 | -0.83 |
| NDUFA6 | NADH:ubiquinone oxidoreductase subunit 6A | 0 | 0 |
| NDUFB2 | NADH:ubiquinone oxidoreductase subunit B2 | -0.55 | 0 |
| NDUFS3 | NADH dehydrogenase [ubiquinone] iron-sulfur protein 3 | 0 | 0 |
| NDUFS5 | NADH:ubiquinone oxidoreductase subunit S5 | 0 | 0 |
| COX11 | Cytochrome c oxidase copper chaperone | 0.61 | -0.33 |
| NDUFV3 | NADH:ubiquinone oxidoreductase subunit V3 | -0.29 | 0 |
| NDUFB9 | NADH:ubiquinone oxidoreductase subunit B9 | 0 | 0.29 |
| ATP50 | ATP synthase, H+ transporting mitochondrial F1 complex, O subunit | -0.55 | 0 |
| NDUFA9 | NADH:ubiquinone oxidoreductase subunit A9 | 0 | 0 |
| CYC1 | Cytochrome C1 | -0.46 | 0 |
| NDUFC1 | NADH:ubiquinone oxidoreductase subunit C1 | -0.43 | 0 |
