# Supplementary material for: Transcriptomic Analyses Reveal Differential Gene Expression of Immune and Cell Death Pathways in the Brains of Mice Infected with West Nile Virus and Chikungunya Virus
Source: Front Microbiol. 2017 Aug 17;8:1556. doi: 10.3389/fmicb.2017.01556 (PMC5562671; doi:10.3389/fmicb.2017.01556)
Supplement: Supplementary file 6 [file Table6.DOCX]

**Table S6.** Differential expression of genes involved in neuronal dysfunction at the late stage of WNV and CHIKV infection compared to early.

| **Neuronal dysfunction** | | **WNV-L vs WNV-E** | **CHIKV-L vs CHIKV-E** |
| --- | --- | --- | --- |
| **Symbol** | **Entrez Gene Name** | **Log_2_ ratio fold change** | **Log_2_ ratio fold change** |
| NMDAR | N-methyl-D-aspartate receptor | -0.45 | 0 |
| GRIA1 | Glutamate ionotropic receptor AMPA type subunit 1 | 0 | 0 |
| GRIA2 | Glutamate ionotropic receptor AMPA type subunit 2 | 0.59 | 0 |
| GRIA3 | Glutamate ionotropic receptor AMPA type subunit 3 | 0 | 0 |
| GRIA4 | Glutamate ionotropic receptor AMPA type subunit 4 | 0 | -0.84 |
| PDGF-B | Platelet-derived growth factor | 0 | 0 |
| VGCC | Voltage-gated calcium channel | - | - |
| FOS | Fos proto-oncogene | 1.18 | 0.74 |
| FOSB | FBJ murine osteosarcoma viral oncogene homolog B | 0.51 | 0 |
| CPG15 | Candidate plasticity gene 15 protein | 0 | 0 |
| CPG16 | Candidate plasticity gene 16 protein | - | - |
| CPG2 (Syne1) | Carboxypeptidase G2 precursor | 0.89 | 0 |
| BDNF | Brain derived neurotrophic factor | 0 | -0.81 |
| TRKB (NTRK2) | Tropomyosin receptor kinase B | -1.14 | -0.69 |
| NTF4 | Neurotrophin 4 | 0 | 0 |
| PLK2 | Polio like kinase 2 | 0.79 | 0 |
| HOMER1 | Homer protein homolog 1 | 1.41 | -0.98 |
| SPARC | Secreted protein acidic and rich in cysteine | -0.22 | -0.61 |
| ARC | Activity-regulated cytoskeleton-associated protein | 1.21 | -0.58 |
| miR-134 | MicroRNA 134 | - | - |
| LIMK1 | LIM domain kinase 1 | -0.55 | 0 |
| CREB1 | CAMP responsive element binding protein 1 | 0 | 0.19 |
| SIK1 | Salt inducible kinase 1 | 0 | 0 |
| RPS6KB1 (RSK) | Ribosomal s6 kinase | 0 | 0 |
| WNT1 | Wnt family member 1 | 0 | 0 |
| CREBBP | CREB-binding protein | 0.48 | -0.40 |
| PER1 | Period circadian protein homolog 1 | 0.72 | 0.77 |
| SS18L1 | Synovial sarcoma translocation gene on chromosome 18-like 1 | 0 | -0.57 |
| NEUROD2 | Neuronal differentiation 2 | 0 | 0 |
| SRE | Serum response element | - | - |
| SRF | Serum response factor | 0 | 0 |
| ELK1 | ETS domain-containing protein Elk-1 | 0 | 0.44 |
| ERK | Extracellular signal–regulated kinases | - | - |
| RhoA | Ras homolog gene family, member A | -0.22 | 0 |
| Rac1 | Ras-related C3 botulinum toxin substrate 1 | -0.21 | 0 |
| Cdc42 | Cell division control protein 42 homolog | 0 | 0.28 |
| USF1 | Upstream stimulatory factor 1 | 0 | 0.58 |
| CARF | Calcium responsive transcription factor | 0 | 0 |
| MECP2 | Methyl CpG binding protein 2 | 0 | -0.46 |
| HDAC1 | Histone deacetylase 1 | 0 | 0 |
| APOB | Apolipoprotein B | 0 | 0 |
| A1CF | APOBEC1 complementation factor | 0 | 0 |
| MEF2C | Myocyte enhancer factor 2C | 0 | 0 |
| CAMK2A | Calcium/calmodulin dependent protein kinase II alpha | 0 | 0.33 |
| NGF | Nerve growth factor | 0 | 0 |
| NFAT | Nuclear factor of activated T-cells | - | - |
| BAD | Bcl-2-associated death promoter | 0 | 0 |
| EGR1 | Early growth response protein 1 | 0.70 | 0 |
| BTG2 | B-cell translocation gene 2 | 0.50 | 0.66 |
| tPA (PLAT) | Tissue plasminogen activator | 1.68 | 0.30 |
| RGS2 | Regulator of G-protein signaling 2 | 0 | -0.55 |
| RHEB | Ras homolog enriched in brain | 0 | 0 |
| PTGS2 | Prostaglandin-endoperoxide synthase 2 | 2.67 | 2.33 |
| NPTX2 | Neuronal pentraxin 2 | 0.46 | 0 |
| PCDH8 | Protocadherin 8 | 0 | 0 |
| MYH1 | Myosin heavy chain 1 | 0 | 0 |
| GABARAP | Gamma-aminobutyric acid receptor-associated protein | 0 | 0 |
| GRM5 | Glutamate metabotropic receptor 5 | -0.30 | 0 |
| MAPT | Microtubule-associated protein tau | -0.43 | -0.29 |
| CHMP2B | Charged multivesicular body protein 2B | -0.26 | 0 |
| GRN | Granulin | 0 | 1.42 |
| GRM3 | Glutamate metabotropic receptor 3 | -1.12 | -0.54 |
| PDGFRL | Platelet derived growth factor receptor like | 0 | 0.27 |
| DAP | Death-associated protein | -0.22 | 0 |
| FOSL2 | FOS like antigen 2 | 0.99 | 0 |
| HOMER2 | Homer scaffolding protein 2 | -0.62 | -0.68 |
| GABRA1 | Gamma-aminobutyric acid (GABA) A receptor, alpha 1 | 0 | 0 |
| CREB3 | CAMP responsive element binding protein 3 | 0.49 | 0 |
| BRSK1 | BR serine/threonine kinase 1 | 0 | 0 |
| CPB1 | Carboxypeptidase B1 | 0 | 0 |
| USF2 | Upstream transcription factor 2 | 0 | 0 |
