# Supplementary material for: Transcriptomic Analyses Reveal Differential Gene Expression of Immune and Cell Death Pathways in the Brains of Mice Infected with West Nile Virus and Chikungunya Virus
Source: Front Microbiol. 2017 Aug 17;8:1556. doi: 10.3389/fmicb.2017.01556 (PMC5562671; doi:10.3389/fmicb.2017.01556)
Supplement: Supplementary file 7 [file Table7.DOCX]

**Table S7.** Differential expression of genes involved in apoptosis at the late stage of WNV and CHIKV infection compared to early.

| **Apoptosis** | | **WNV-L vs WNV-E** | **CHIKV-L vs CHIKV-E** |
| --- | --- | --- | --- |
| **Symbol** | **Entrez Gene Name** | **Log_2_ ratio fold change** | **Log_2_ ratio fold change** |
| AATF | Apoptosis antagonizing transcription factor | 0.43 | 0 |
| ABL1 | ABL proto-oncogene 1, non-receptor tyrosine kinase | 0 | 0 |
| AKT1 | AKT serine/threonine kinase 1 | -0.43 | 0 |
| APAF1 | Apoptotic peptidase activating factor 1 | 0.50 | 0 |
| ATP6V1G2 | ATPase H+ transporting V1 subunit G2 | -0.22 | 0 |
| BAX | BCL2 associated X | 0 | 0 |
| BCL2 | BCL2, apoptosis regulator | 0.83 | -0.70 |
| BCL2A1 (Bfl-1) | BCL2 related protein A1 | 2.26 | 3.24 |
| BCL2L1 | BCL2 like 1 | -0.17 | 0.40 |
| BCL2L11 | BCL2 like 11 | 0.48 | 0.73 |
| BFAR | Bifunctional apoptosis regulator | 0 | 0 |
| BIRC2 (cIAP2) | Baculoviral IAP repeat containing 2 | 1.09 | 0.44 |
| BIRC3 (cIAP1) | Baculoviral IAP repeat containing 3 | 2.05 | 1.65 |
| CASP12 | Caspase 12 | 1.52 | 1.44 |
| CASP14 | Caspase 14 | -0.27 | 0 |
| CASP2 | Caspase 2 | 0.34 | 0 |
| CASP3 | Caspase 3 | 0 | -0.34 |
| CASP6 | Caspase 6 | 0 | -0.36 |
| CASP8 | Caspase 8 | 0.94 | 0.68 |
| CASP9 | Caspase 9 | 0 | 0 |
| CD40 (TNFRSF5) | Cluster of differentiation-40 | 0.94 | 1.27 |
| CD40LG (TNFSF5) | Cluster of differentiation-40 ligand | -0.25 | 0 |
| CFLAR (CASPER) | CASP8 and FADD like apoptosis regulator | 1.40 | 1.43 |
| CYLD | CYLD lysine 63 deubiquitinase | 0 | 0 |
| DFF40 (CAD) | Carbamoyl-phosphate synthetase 2, aspartate transcarbamylase, and dihydroorotase | -0.48 | 0 |
| DFF45 (DFFA) | DNA fragmentation factor subunit alpha | -0.16 | 0 |
| FADD | Fas associated via death domain | 0 | 0 |
| FAS (TNFRSF6) | Fas cell surface death receptor | 1.61 | 1.27 |
| FASLG (TNFSF6) | Fas ligand | 0.59 | 0 |
| GADD45A | Growth arrest and DNA damage inducible alpha | 1.45 | 0 |
| IGF1R | Insulin like growth factor 1 receptor | 0 | 0 |
| IL15 | Interleukin 15 | 1.30 | 1.09 |
| IL15RA | Interleukin 15 receptor subunit alpha | 1.19 | 1.02 |
| MCL1 | BCL2 family apoptosis regulator | 1.04 | 1.01 |
| MMP2 | Matrix metallopeptidase 2 | -0.25 | 0 |
| MMP3 | Matrix metallopeptidase 3 | 0.38 | 0.47 |
| MMP9 | Matrix metallopeptidase 9 | 0 | 0 |
| NAIP2 | NLR family apoptosis inhibitory protein 2 | 0.69 | 0.48 |
| NAIP5 | NLR family apoptosis inhibitory protein 5 | 0 | 0 |
| NOL3 | Nucleolar protein 3 | 0 | 0 |
| SPATA2 | Spermatogenesis associated 2 | 0.28 | 0 |
| SYCP2 | Synaptonemal complex protein 2 | 0.53 | 0 |
| TNF | Tumor necrosis factor | 1.15 | 0.73 |
| TNFRSF10A (TRAIL-R) | TNF receptor superfamily member 10a | 0 | 0 |
| TNFRSF10B (TRAIL-R2) | TNF receptor superfamily member 10b | 0 | 0 |
| TNFRSF11B | TNF receptor superfamily member 11b | 1.11 | 0 |
| TNFRSF1A | TNF receptor superfamily member 1a | 0.77 | 1.15 |
| TNFSF10 (TRAIL) | Tumor necrosis factor superfamily member 10 | 0.96 | 1.33 |
| TP53 | Tumor protein p53 | 0.32 | 0.47 |
| TRADD | Tumor necrosis factor receptor type 1-associated DEATH domain | -0.18 | 0 |
| TRAF2 | TNF receptor-associated factor 2 | 0.30 | 0.52 |
| XIAP (BIRC4) | X-linked inhibitor of apoptosis protein | 0.39 | 0.23 |
| TNFRSF1B | TNF receptor superfamily member 1b | 0.70 | 1.03 |
| PMAIP1 | Phorbol-12-myristate-13-acetate-induced protein 1 | 0.97 | 0.50 |
| CYCS | Cytochrome c somatic | -0.30 | 0.27 |
| HRK (DP5) | Harakiri, BCL2 Interacting Protein | 0.39 | 0.35 |
| CD36 | Cluster of differentiation-36 | 0 | 0.61 |
| ANXA1 | Annexin A1 | 0.80 | 0.99 |
| BAD | BCL2 associated agonist of cell death | 0 | 0 |
| BAK1 | BCL2 antagonist/killer 1 | 0.35 | 0.50 |
| BBC3 | BCL2 binding component 3 | 0.48 | 0 |
| BCL2L2 | BCL2 like 2 | 0.24 | -0.35 |
| BID | BH3 interacting domain death agonist | 1.01 | 0.92 |
| BIK | BCL2 interacting killer | 0 | 0 |
| BMF | Bcl2 modifying factor | 0 | 0.27 |
| BNIP3 | BCL2 interacting protein 3 | 0 | 0 |
| BNIP3L | BCL2 interacting protein 3 like | 0.28 | 0 |
| CALR | Calreticulin | -0.62 | 0 |
