# Supplementary material for: Transcriptomic Analyses Reveal Differential Gene Expression of Immune and Cell Death Pathways in the Brains of Mice Infected with West Nile Virus and Chikungunya Virus
Source: Front Microbiol. 2017 Aug 17;8:1556. doi: 10.3389/fmicb.2017.01556 (PMC5562671; doi:10.3389/fmicb.2017.01556)
Supplement: Supplementary file 8 [file Table8.DOCX]

**Table S8.** Differential expression of genes involved in pyroptosis at the late stage of WNV and CHIKV infection compared to early.

| **Pyroptosis** | | **WNV-L vs WNV-E** | **CHIKV-L vs CHIKV-E** |
| --- | --- | --- | --- |
| **Symbol** | **Entrez Gene Name** | **Log_2_ ratio fold change** | **Log_2_ ratio fold change** |
| AIM2 | Absent in melanoma 2 | 0 | 0 |
| CARD6 | Caspase recruitment domain family member 6 | 1.28 | 0 |
| CASP1 | Caspase 1 | 0.99 | 1.51 |
| CASP4 | Caspase 4 | 2.34 | 2.66 |
| CASP7 | Caspase 7 | 0.41 | 0.58 |
| CASP11 | Caspase 11 | - | - |
| DDX10 | DEAD-box helicase 10 | 0.50 | 0 |
| DDX18 | DEAD-box helicase 18 | 0.40 | 0 |
| DDX24 | DEAD-box helicase 24 | 0 | 0.36 |
| DDX39A | DEAD-box helicase 39A | 0.76 | 0 |
| DDX49 | DEAD-box helicase 49 | 0 | 0.36 |
| DDX54 | DEAD-box helicase 54 | 0 | 0 |
| DDX6 | DEAD-box helicase 6 | 0.87 | 0 |
| GSDMD | Gasdermin D | 0 | 0.95 |
| IL18 | Interleukin 18 | 0 | 0 |
| IL1B | Interleukin 1B | 0.75 | 1.43 |
| LRR1 | Leucin rich repeat protein 1 | -0.39 | 0 |
| LRRC16 (CARMIL1) | Capping protein regulator and myosin 1 linker 1 | 0.61 | 0 |
| LRRC33 (NRROS) | Negative regulator of reactive oxygen species | 0 | 0.64 |
| LRRC4B | Leucine rich repeat containing 4B | - | - |
| LRRC4C | Leucine rich repeat containing 4C | 0 | 0 |
| LRRC58 | Leucine rich repeat containing 58 | 0 | 0 |
| LRRTM1 | Leucine rich repeat transmembrane neuronal 1 | 0 | 0.37 |
| MDA5 (IFIH1) | Interferon induced with helicase C domain 1 | 0.80 | 2.65 |
| NLRC3 | NLR family CARD domain containing 3 | 0 | -0.42 |
| NLRC4 (IPAF) | NLR family CARD domain containing 4 | - | - |
| NLRC5 | NLR family CARD domain containing 5 | 1.14 | 2.54 |
| NLRP1 | NLR family pyrin domain containing 1 | - | - |
| NLRP3 | NLR family pyrin domain containing 3 | 0 | 0.74 |
| NOD1 | Nucleotide binding oligomerization domain containing 1 | 1.00 | 0.98 |
| NOD2 | Nucleotide binding oligomerization domain containing 2 | - | - |
| PYCARD (ASC) | PYD and CARD domain containing | 0.80 | 1.34 |
| RIG-I (DDX58) | DExD/H-box helicase 58 | -0.35 | 2.01 |
