# Supplementary material for: Transcriptomic Analyses Reveal Differential Gene Expression of Immune and Cell Death Pathways in the Brains of Mice Infected with West Nile Virus and Chikungunya Virus
Source: Front Microbiol. 2017 Aug 17;8:1556. doi: 10.3389/fmicb.2017.01556 (PMC5562671; doi:10.3389/fmicb.2017.01556)
Supplement: Supplementary file 9 [file Table9.DOCX]

**Table S9.** Differential expression of genes involved in autophagy at the late stage of WNV and CHIKV infection compared to early.

| **Autophagy** | | **WNV-L vs WNV-E** | **CHIKV-L vs CHIKV-E** |
| --- | --- | --- | --- |
| **Symbol** | **Entrez Gene Name** | **Log_2_ ratio fold change** | **Log_2_ ratio fold change** |
| AKT1 | AKT serine/threonine kinase 1 | -0.43 | 0 |
| AMBRA1 | Autophagy and beclin 1 regulator 1 | 0 | 0 |
| AMPK | [AMP-activated protein kinase](https://en.wikipedia.org/wiki/AMP-activated_protein_kinase) | - | - |
| APP | Amyloid precursor protein | 0 | 0.27 |
| ATG101 | Autophagy related 101 | 0 | 0 |
| ATG12 | Autophagy related 12 | 0.45 | 0 |
| ATG13 | Autophagy related 13 | -0.16 | 0 |
| ATG14 | Autophagy related 14 | 0.55 | 0 |
| ATG16L1 | Autophagy-related 16-like 1 | 0.47 | 0 |
| ATG3 | Autophagy related 3 | 0 | 0 |
| ATG7 | Autophagy related 7 | -0.34 | 0 |
| ATOH7 | Atonal BHLH transcription factor 7 | 0 | 0 |
| BAX | BCL2 associated X | 0 | 0 |
| BCL2 | BCL2, apoptosis regulator | 0.83 | -0.70 |
| BCL2L1 | BCL2 like 1 | -0.17 | 0.40 |
| BECN1 | Beclin 1 | 0 | 0 |
| CYCS | Cytochrome C somatic | -0.30 | 0.27 |
| BNIP3 | BCL2 interacting protein 3 | 0 | 0 |
| CAPN1 | Calpain 1 | 0 | 0 |
| CTSB | Cathepsin B | 0.86 | 0.74 |
| CTSS | Cathepsin S | 1.12 | 0.72 |
| DRAM1 | DNA damage regulated autophagy modulator 1 | 1.26 | 0.74 |
| ESR1 (Era) | Estrogen receptor 1 | 0 | 0 |
| FADD | Fas associated via death domain | 0 | 0 |
| FAS (TNFRSF6) | Fas cell surface death receptor | 1.61 | 1.27 |
| GAA | Glucosidase alpha, acid | 0 | 0 |
| HIP2 (UBE2K) | Ubiquitin conjugating enzyme E2 K | 0.19 | 0 |
| IFNG | Interferon gamma | 2.56 | 0.57 |
| IGF1 | Insulin-like growth factor 1 | 0 | -0.61 |
| INS1 | Insulin 1 | -0.21 | 0 |
| INS2 | Insulin 2 | 0 | 0 |
| IRGM2 | Immunity-related GTPase family M member 2 | 1.08 | 1.62 |
| MAP1LC3A | Microtubule associated protein 1 light chain 3 alpha | -0.70 | 0.36 |
| MAPK8 (JNK1) | Mitogen-activated protein kinase 8 | 0.36 | 0 |
| MTOR (FRAP) | Mechanistic target of rapamycin | -0.51 | 0 |
| NFKB1 | Nuclear factor kappa B subunit 1 | 0.77 | 0 |
| PIK3C3 (VPS34) | Phosphatidylinositol 3-kinase catalytic subunit type 3 | 0 | 0 |
| RB1CC1 (FIP200) | RB1 inducible coiled-coil 1 | 0.45 | 0 |
| RPS6KB1 | Ribosomal protein S6 kinase B1 | 0 | 0 |
| RUBCN | RUN and cysteine rich domain containing Beclin 1 interacting protein | - | - |
| SNCA | Synuclein alpha | 0 | 0 |
| SQSTM1 | Sequestosome 1 | 0.54 | 0.34 |
| TNF | Tumor necrosis factor | 1.15 | 0.73 |
| TP53 | Tumor protein p53 | 0.32 | 0.47 |
| TNFSF10 (TRAIL) | Tumor necrosis factor superfamily member 10 | 0.96 | 1.33 |
| ULK1 | Unc-51 like autophagy activating kinase 1 | 0 | 0 |
| UVRAG | UV radiation resistance associated | 0.71 | 0 |
